# Liposome-Templated Indocyanine Green J- Aggregates for *In Vivo* Near-Infrared Imaging and Stable Photothermal Heating

**DOI:** 10.7150/ntno.41737

**Published:** 2020-02-28

**Authors:** Calvin C. L. Cheung, Guanglong Ma, Kostas Karatasos, Jani Seitsonen, Janne Ruokolainen, Cédrik-Roland Koffi, Hatem A.F.M Hassan, Wafa' T. Al-Jamal

**Affiliations:** 1 School of Pharmacy, Queen's University Belfast, Belfast, BT9 7BL, United Kingdom; 2 Department of Chemical Engineering, University of Thessaloniki, P.O. BOX 420, 54124 Thessaloniki, Greece; 3 Department of Applied Physics, Aalto University School of Science, P.O.Box 15100, FI-00076 Aalto, Finland

**Keywords:** J-aggregates, indocyanine green, liposomes, theranostics, photothermal therapy

## Abstract

Indocyanine green (ICG) is an FDA-approved near-infrared fluorescent dye that has been used in optical imaging and photothermal therapy. Its rapid *in vivo* clearance and photo-degradation have limited its application. ICG pharmacokinetics and biodistribution have been improved via liposomal encapsulation, while its photothermal stability has been enhanced by ICG J-aggregate (IJA) formation. In the present work, we report a simple approach to engineer a nano-sized, highly stable IJA liposomal formulation. Our results showed that lipid film hydration and extrusion method led to efficient IJA formation in rigid DSPC liposomes, as supported by molecular dynamics modeling. The engineered DSPC-IJA formulation was nano-sized, and with spectroscopic and photothermal properties comparable to free IJA. Promisingly, DSPC-IJA exhibited high fluorescence, which enabled its* in vivo* tracking, showing prolonged blood circulation and significantly higher tumor fluorescence signals, compared to free ICG and IJA. Furthermore, DSPC-IJA demonstrated high photo-stability *in vivo* after multiple cycles of 808 nm laser irradiation. Finally, doxorubicin was loaded into liposomal IJA to utilize the co-delivery capabilities of liposomes. In conclusion, with both liposomes and ICG being clinically approved, our novel liposomal IJA could offer a clinically relevant theranostic platform enabling multimodal imaging and combinatory chemo- and photothermal cancer therapy.

## Introduction

Indocyanine green (ICG) is a near-infrared (NIR) fluorescent dye (marketed as IC-Green^®^), and has been clinically applied for angiography examination, sentinel lymph node mapping, and intraoperative imaging 1. ICG has been also used as a photoacoustic agent 2, photothermal agent 3, and photosensitizer 4. ICG is rapidly bound to plasma proteins *in vivo* and cleared from the circulatory system 5, and exhibits inherent low photo-, thermal- and aqueous-stability 6-8, limiting its development as a therapeutic agent. To overcome these limitations, ICG has been encapsulated in nanocarriers such as inorganic nanoparticles 9, polymeric nanoparticles 10 and liposomes 2,3,11. Liposomes are particularly attractive for their clinically well-established pharmacokinetics, pharmacodynamics, and biocompatibility 12-14. More importantly, liposomal encapsulation has improved ICG stability, fluorescence quantum yield, photothermal conversion efficiency 3,11.

ICG photo-degradation, through the oxidative cleavage of the polymethine chain, remained unavoidable unless chemical modifications are made 15. Unfortunately, ICG rapid photo-degradation upon irradiation hampers its development as an efficient photothermal agent 3,16. J-aggregates formation is an alternative approach to improve ICG overall stability 17,18. In 1936, E. E. Jelley (hence the letter J in J-aggregate) discovered the existence of nematic (almost parallel) aggregates of the dye pseudoisocyanine chloride, exhibiting distinctive spectroscopic properties 19. J-aggregates are characterized by absorption spectra with a very narrow width (10 - 20 nm), dramatic redshift (~ 100 nm), and strongly increased molar attenuation coefficient, in comparison with their monomers. Their fluorescence emission spectra are also redshifted and sharpened, with a dramatic reduction in radiative lifetime, termed “superradiance” 17,18. As a result, J-aggregates of several organic dyes are spectroscopically active in the NIR region, allowing bioimaging with minimal auto-fluorescence from biological tissues, reduced light scattering, and higher tissue penetration 18. ICG J-aggregate (IJA), first reported more than 50 years ago, demonstrated exceptional photo-, thermal-, and aqueous-stability compared to monomeric ICG 6,20. Encouragingly, Liu et al. recently reported IJA as a theranostic agent enabling both fluorescence/optoacoustic imaging and photothermal therapy *in vivo* 21.

Despite the long history of both liposomes 22 and IJA 20, a liposomal formulation of IJA has not been reported yet. Combining the advantages of liposomes and IJA could result in a promising theranostic agent effectively uniting NIR fluorescence/optoacoustic imaging and photothermal property of ICG with liposomes' biocompatibility, superior pharmacokinetics and biodistribution. In the present work, we report the development of a liposomal IJA formulation, which was prepared in situ using lipid film hydration and extrusion. Factors influencing IJA formation in liposomes, namely ICG concentration, incubation temperature, membrane rigidity, and hydration medium were investigated. We also characterized the liposomal IJA size, dispersity, stability, and morphology. Liposome- templated IJA formation using different lipid bilayers was also studied using molecular dynamics modeling. Finally, the fluorescence imaging and photothermal heating capabilities of liposomal IJA were evaluated *in vivo*.

## Experimental Section

### Materials

1,2-dioleoyl-sn-glycero-3-phosphatidylcholine (DOPC), 1,2-dipalmitoyl-sn-glycero-3-phosphatidylcholine (DPPC), 1,2-distearoyl-sn-glycero-3-phosphatidylcholine (DSPC), 1,2-distearoyl-sn-glycero-3-phosphoethanolamine-N-[methoxy(polyethylene glycol)-2000] (DSPE-PEG_2000_), were generous gifts from Lipoid GmbH (Ludwigshafen, Germany). Dimethyl sulfoxide (DMSO) was purchased from Alfa Aesar (Lancashire, UK). Ammonium sulfate ((NH_4_)_2_SO_4_), cholesterol (Chol), dextrose, 4-(2-hydroxyethyl)-1-piperazineethanesulfonic acid (HEPES), indocyanine green (ICG), phosphate buffered saline (PBS) tablets, sodium chloride (NaCl), were purchased from Sigma-Aldrich Ltd. (Dorset, UK). Doxorubicin hydrochloride (DOX) was purchased from Apollo Scientific (Cheshire, UK). Deionized water (DW; Ultrapure water, 18.2 MΩ) was used throughout the experiment. All chemicals were used without further purification.

### Preparation of ICG J-Aggregate (IJA)

ICG J-aggregate (IJA) was prepared by heat treatment of ICG aqueous solution 21,23. ICG solution in DW (645 µM; 0.5 mg mL^-1^) was heated at 65 °C for 32 h. The solution was dialyzed against DW for 24 h using Pur-A-Lyzer™ Dialysis Kit (12 kDa molecular weight cut-off; Sigma-Aldrich, Dorset, UK) and then stored at -20 °C.

### Preparation of liposomal ICG and IJA

Liposomes composed of DOPC/Chol/DSPE- PEG_2000_ (95/50/5 molar ratio), DPPC/Chol/DSPE- PEG_2000_ (95/50/5 molar ratio), and DSPC/Chol/ DSPE-PEG_2000_ (95/50/5 molar ratio) were prepared by lipid film hydration followed by extrusion. Organic solvents were removed by rotary evaporation (BUCHI Labortechnik AG, Flawil, Switzerland) at 60 °C, then dried lipid film was hydrated with a hydrating medium at 60 °C for 30 min, to achieve a final lipid and ICG concentration of 7.5 mM (5 mM phospholipid and 2.5 mM cholesterol) and 180 µM, respectively. For some initial experiments, ICG concentration ranged between 30 - 180 µM. The different hydrating media used in this work were HEPES-buffered saline (HBS; 20 mM HEPES, 137 mM NaCl, pH 7.4), DW, (NH_4_)_2_SO_4_ (240 mM, pH 5.4) and dextrose solution (DEX; 5% w/v). Hydrated liposome suspension was extruded through polycarbonate membranes (0.8 µm, 7 times; 0.2 µm, 11 times; 0.1 µm, 15 times) at 60 °C using the Avanti^®^ mini-extruder (Avanti Polar Lipids Inc., AL, USA). Extruded liposomes were left to anneal at 65 °C for 30 min, then purified by size-exclusion chromatography with PD-10 desalting columns prepacked with Sephadex™ G-25 resins (GE Healthcare Life Sciences, Buckinghamshire, UK) using HBS buffer.

### Size characterization of liposomal ICG and IJA

Z-average diameter and dispersity were determined using Zetasizer Nano ZS90 (Malvern Panalytical, Worcestershire, UK) equipped with a 4.0 mW He-Ne laser operating at 633 nm with photodiode detector at a detection angle at 90°. Liposomal samples were diluted 10-fold in DW and transferred to low-volume polystyrene cuvettes. Z-average and dispersity of each sample were obtained as the mean of three measurements.

### Determination of ICG and IJA optical properties and encapsulation efficiencies (ICG EE)

ICG and IJA optical properties and ICG encapsulation efficiencies (ICG EE) of liposomes were determined by measuring their absorbance using FLUOstar Omega Microplate Reader (BMG Labtech, Buckinghamshire, UK) with a resolution of 1 nm. IJA-to-ICG absorbance ratio (IJA/ICG) was taken as the ratio of absorbance at 892 nm to 792 nm (i.e. A_892_/A_792_ ratio). A calibration curve of monomeric ICG (0.3125 - 10 µM) was established in HBS/DMSO (1/4, v/v) at an absorbance wavelength of 792 nm, corresponding to monomeric ICG. Quantification of loaded ICG was performed by solubilizing the purified liposomes in HBS/DMSO (1/4, v/v) and interpolated using the calibration curve. ICG EE was determined by taking the ratio between the concentration of encapsulated ICG and initial ICG concentration.


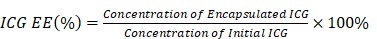
(1)

### Cryogenic transmission electron microscopy (Cryo-TEM)

5 µL of the sample was deposited on Quantifoil R 2/1 200 mesh holey carbon-coated copper grids. The excess solution was removed by blotting for 3 s in 80% relative humidity using an automatic plunge freezer (EM GP2, Leica Microsystem), followed by immediate vitrification using liquid ethane (-175 °C). Vitrified samples were cryo-transferred to the microscope and imaged using a JEOL JEM-3200 FSC TEM while maintaining specimen temperature at -187 °C.

### Molecular dynamics (simulation of the ICG and lipid bilayer)

Fully atomistic molecular dynamic simulations were performed on hydrated DOPC/Chol/DSPE- PEG_2000_ (DOPC), DPPC/Chol/DSPE-PEG_2000_ (DPPC), and DSPC/Chol/DSPE-PEG_2000_ (DSPC) bilayers, constructed with the aid of the CHARMM-GUI 24 and the Packmol program 25, with molar ratios close to the experimental conditions (i.e., 100/50/5, molar ratio). Each lipid bilayer comprised 240 phospholipids, 120 cholesterol, 12 DSPE-PEG_2000_, 15 ICG molecules hydrated by a number of water molecules close to 100000, which corresponded to an ICG concentration of about 7 mM. In the initial configurations, ICG molecules were placed in random orientations and positions, on the ambilateral of the lipid bilayer, with a maximum distance of approximately 60 Å from the bilayer's surface. A control system comprised 15 ICG molecules, and water (ICG-water) was also examined. The systems were simulated at 60 °C.

All simulations were performed using the NAMD 2.13 package 26 in the isothermal-isobaric ensemble (*p* = 100 kPa), with energetic parameters based on the general AMBER force field (GAFF) 27,28. Production runs of 50 ns were generated and analyzed.

### DOX loading into liposomal IJA using the pH-gradient remote loading method

DOX was loaded into liposomes using a pH-gradient remote loading method. Liposomes were first prepared in (NH_4_)_2_SO_4_ as the aqueous medium as described previously. Following purification by size-exclusion chromatography, the liposomes were incubated with DOX at drug-to-phospholipid (excluding cholesterol) molar ratio of 1:20 at 60 °C for 1 h. After incubation, liposomes were purified by removing unencapsulated DOX using size-exclusion chromatography, as described above. To quantify the encapsulation efficiency (%EE) of DOX, liposomes before and after purification were diluted to the same lipid concentration and then solubilized by Triton X-100 to release encapsulated DOX. A final concentration of 0.1 v/v % Triton X-100 was used, corresponds to a phospholipid-to-detergent molar ratio of 1:20, sufficient to ensure complete solubilization of liposomes 29. DOX fluorescence intensity was measured using a microplate reader with excitation and emission wavelength of 485 nm and of 590 nm, respectively. The concentration of DOX in the wells were within the linear region. DOX %EE was then calculated by comparing the fluorescence intensity of the samples before and after purification:


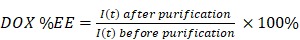
(2)

### Fluorescence imaging

Samples containing ICG (0.2 mL, 2.58 µM; 2 µg mL^-1^) were dispersed in HBS or HBS/DMSO (1/4, v/v). Samples were imaged by the Bruker InVivo Xtreme Imaging System (Bruker Scientific LLC, MA, USA) with an exposure time of 0.2 s, using excitation and emission wavelengths of 760 nm and 830 nm, respectively.

### Near-Infrared (NIR) laser-induced photothermal heating

NIR laser-induced photothermal heating was performed using FC-808 fiber-coupled laser system (CNI Optoelectronics Tech, Changchun, China) operating at a laser wavelength of 808 nm and power of 0.5 W cm^-2^. Assessing the IJA photothermal stability using 900 nm laser could not be studied, since accessing 900 nm laser was not possible. Samples containing ICG (0.5 mL, 12.9 µM; 10 µg mL^-1^) were placed in a transparent polystyrene cuvette in a customized holder to maintaining the temperature of the samples at 36 ºC, using a peristaltic pump (Verderflex, Castleford, UK) and water bath (Grant Instruments Ltd., Royston, UK). Samples were irradiated for 300 s then cooled down for 300 s; three cycles were performed. The temperature was monitored with a fiber optic temperature probe (Model PRB-G40, Osensa, Canada).

### *In vivo* fluorescence imaging

5-week-old female BALB/c mice were purchased from Envigo (Bicester, UK) and animal procedures were performed in compliance with the UK Home Office Code of Practice for the Housing and Care of Animals used in Scientific Procedures. Mice were inoculated with 2.5 × 10^5^ CT 26 (murine colon) or 4T1 (murine breast cancer) cells in 20 µL PBS by subcutaneous injection in the right lower leg using 26 G hypodermic needles. C4-2B (human prostate cancer) model was established in 8-10-week old male NSG mice (bred-in-house) by subcutaneous inoculation of the flank with 5 × 10^6^ C4-2B cells in 25 µL scrum-free medium mixed with Corning^®^ Matrigel^®^ Matrix High Concentration (1:1, v/v).

Mice with suitable tumor size were used for *in vivo* fluorescent imaging. All tumor-bearing mice were placed on Teklad Global 2019X food Envigo (Bicester, UK) 5-day prior imaging, and shaved using a hair removal cream. Mice were injected via tail vein equivalent dose of 0.3 mg kg^-1^ (30 µg mL^-1^, 200 µL per mouse; 6 µg ICG equivalence) of ICG, IJA or DSPC-IJA in HBS. Mice were anesthetized with isoflurane, and body temperature were monitored using a heating pad and a rectal thermocouple. Mice were imaged 1, 24 and 48 h post-injection using the Bruker InVivo Xtreme imaging system with an exposure time of 0.2 s, using excitation and emission wavelengths of 760 nm and 830 nm, respectively. At the end of the study, mice were sacrificed, and the organs and tumors were excised and imaged as described above.

### *In vivo* photothermal heating

48 h post-injection, as described above, mice were anesthetized with isoflurane, and body temperature was monitored using a heating pad and a rectal thermocouple. CT26 tumors were irradiated with 808 nm laser at a power of 0.5 W cm^-2^ for 5 min. Tumors temperature was monitored with FLIR C3 thermal camera (FLIR Systems UK, Kent, UK).

### Statistical analysis

Student's unpaired two-tailed t-test and one-way analysis of variance (ANOVA) followed by Fisher's least significant difference (LSD) test were used to assess statistical significance between group means 30,31. All analyses were performed, with the significance level α of 0.05, using GraphPad Prism 7.0 (GraphPad Software Inc., CA, US).

## Results

### IJA strongly absorbs at 892 nm with superior optical stability in high salt-containing media

**Figure 1:**
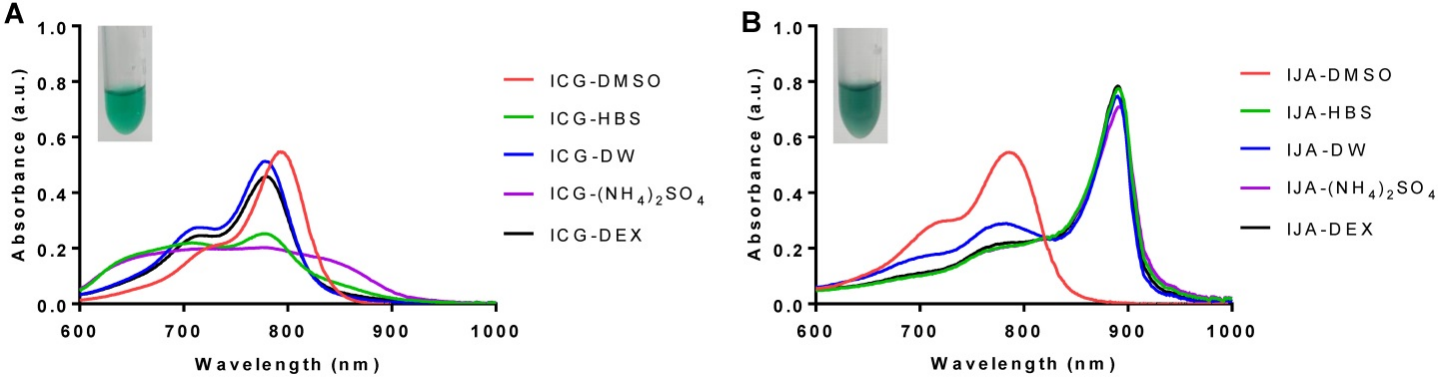
** Absorption spectra of ICG and IJA dispersed in different aqueous media.** 5 µM of (A) ICG and (B) IJA dispersed in DMSO (red), HBS (green), (NH_4_)_2_SO_4_ (blue), DEX (violet) and DW (black). Inset of 100 µM ICG (left) and IJA (right) dispersed in HBS. Data represents mean of at least three independent experiments.

The surrounding environment highly influences ICG optical properties and its aggregation states (H- and J-aggregates) 7,8. In the present study, ICG J-aggregate (IJA) was prepared by 24 h heat treatment of 645 µM (0.5 mg mL^-1^) of ICG solution in deionized water (DW) 23, and its formation was monitored spectroscopically (Figure S1, Supporting Information). To assess the effect of the surrounding environment on ICG and IJA optical properties, 5 µM ICG or IJA were dispersed in various media (**Figure 1**). As expected, ICG was highly soluble in DMSO, demonstrated by a dominant peak of monomeric ICG (792 nm) and a shoulder peak at 715nm (H-aggregate). ICG dispersed in DW exhibited a monomeric peak at 780 nm and a weaker H-aggregate peak at 715 nm. ICG in dextrose solution (DEX) exhibited similar absorbance to that in DW, with a slight reduction in overall absorbance. On the other hand, ICG in HEPES buffered saline (HBS) showed much weaker absorbance with two comparable monomeric and H-aggregate peaks, which has been inferred to the aggregation-promoting effect of NaCl 7,8. Moreover, ICG in (NH_4_)_2_SO_4_ did not show well-defined peaks, rather a broad peak from 700 nm to 850 nm, which might be attributed to the higher ionic strength of (NH_4_)_2_SO_4_ compared to HBS. Collectively, these results are in agreement with previous work, reporting ICG aggregation in salt-containing media 7.

The difference between ICG and IJA was visually distinguishable, with IJA being dark green (Figure 1B, inset) compared to the light green ICG (Figure 1A, inset). IJA exhibited a strong redshift of 100 nm and absorbed strongly at 892 nm, and completely dissociated in DMSO to monomeric ICG (Figure 1B, red line), as reported by others 21. In contrast to ICG, IJA dispersed in different aqueous media displayed similar characteristic absorbance peaks at 892 nm, demonstrating stronger aqueous stability and no observable salt-induced spectroscopic changes. Some monomeric ICG was observed in DW, which may be attributed to the dissociation of IJA in DW at a low concentration 6.

### Liposomal IJA formation is temperature-dependent but ICG is concentration-independent

IJA formation is known to be dependent on temperature, time, and ICG concentration 20,23. In order to explore the possibility of ICG J-aggregation in liposomes, high phase transition temperature DSPC/Chol/DSPE-PEG_2000_ (DSPC) lipid bilayer was first used. DSPC-IJA liposomes in HBS (DSPC- IJA-HBS) were prepared and extruded at 55, 60, and 65 °C to investigate the effect of preparation temperature on IJA formation (Figure S2, Supporting Information). It is worth noting that after lipid film hydration and before membrane extrusion, IJA was barely observed in all DSPC liposomal formulations (Figure S2a, Supporting Information). Interestingly, upon extrusion, IJA was efficiently formed with the corresponding reduction in the H-aggregate and monomeric ICG peaks. The highest IJA formation was observed in the sample prepared at 60 °C, while its extent was lower at 55 and 65 °C, indicating that 60 °C is the optimum temperature for initial liposomal-IJA formation (Figure S2b, Supporting Information). Nonetheless, following the annealing of liposomes at 65 °C for 30 min, greater proportions of J-aggregates were observed (Figure S2c, Supporting Information).

Following optimizing the hydration temperature of liposomes to induce IJA formation, DPSC-IJA-HBS was prepared with a range of initial ICG concentrations of 30, 60, 120, and 180 µM (Figure S3, Supporting Information). DSPC-IJA-HBS liposome size, dispersity, ICG EE and IJA/ICG are listed in **Table 1**. All prepared DSPC-IJA-HBS exhibited a homogenous distribution of small size, ranges between 140 - 170 nm. Larger liposomes were obtained when loading with 180 µM ICG (together with a relatively lower ICG EE), suggested that the concentration loaded is approaching saturation. However, the difference in size due to initial ICG concentration was statistically insignificant (F_4,10_ = 2.968, p > 0.05). The characteristic IJA absorbance peak, at 892 nm, was evident in all DSPC-IJA-HBS, prepared at 60 °C with an initial of 30 to 180 µM of ICG. With increasing initial ICG concentration, despite the decreasing ICG EE, the IJA/ICG increased from 1.379 to 3.292, suggesting that the majority of the ICG loaded into liposomes existed as J-aggregate.

### Liposomal IJA formation is directly proportional to the rigidity of the lipid bilayer

**Figure 2:**
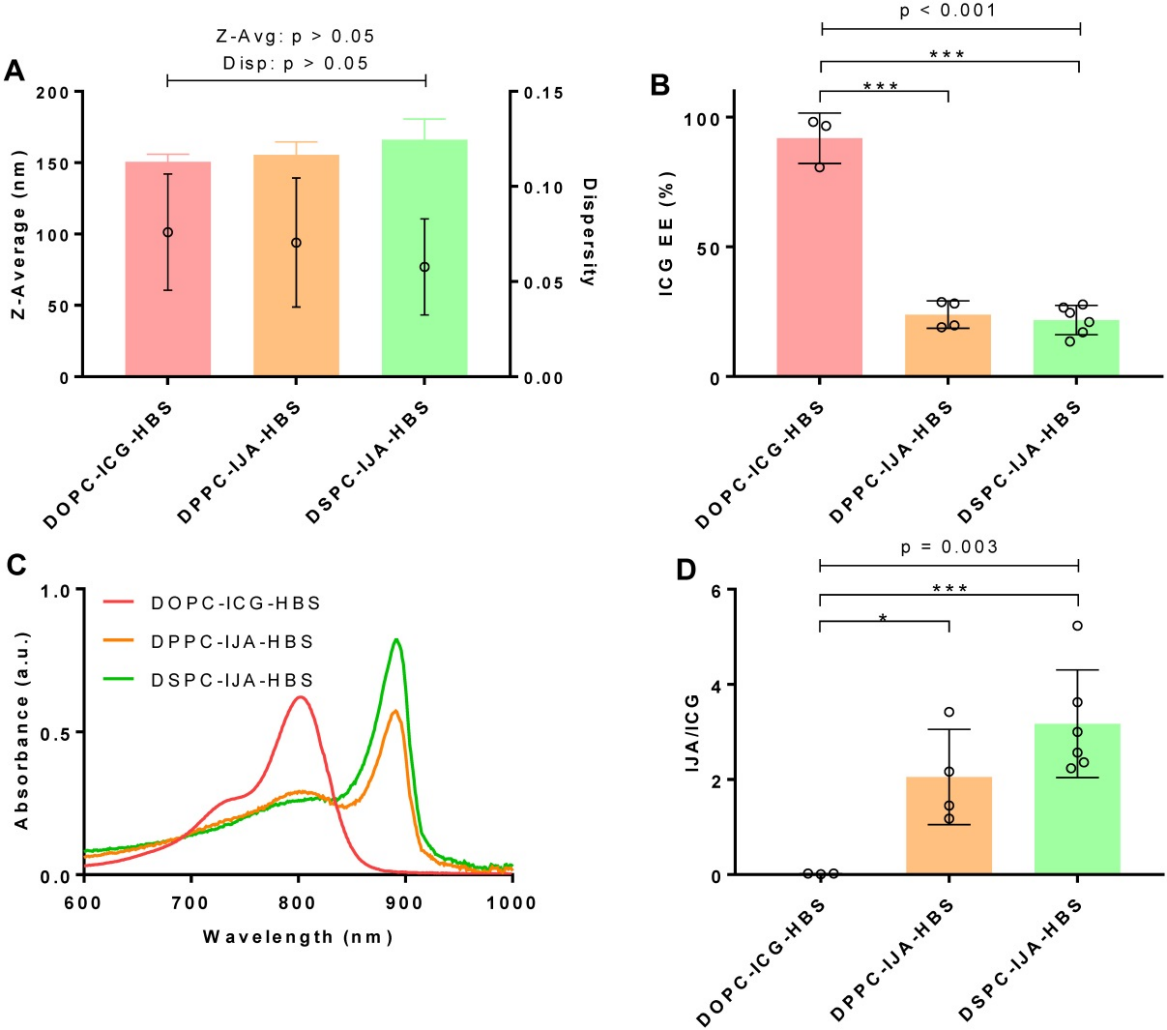
** Characterization of liposomal IJA of different lipid bilayer rigidity.** (A) Z-average diameter and dispersity; (B) ICG EE; (C) absorption spectra; and (D) IJA-to-ICG absorbance ratio of DOPC-ICG-HBS (red), DPPC-IJA-HBS (orange) and DSPC-IJA-HBS liposomes (green) loaded with 180 µM of ICG. Absorption spectra are normalized equivalent to 5 µM of ICG. Data represent mean ± *SD* of at least three independent experiments. *, p < 0.05; ***, p < 0.001; Fisher's LSD test.

To investigate the influence of lipid bilayer on IJA formation, ICG was loaded into lipid bilayer of different membrane rigidity; namely, DOPC (T_m_ = -17 °C), DPPC (T_m_ = 41 °C) and DSPC (T_m_ = 55 °C), using HBS as the aqueous medium, 180 µM ICG, and 60 °C for hydration and extrusion. The cholesterol (50 mol%) and DSPE-PEG_2000_ content (5 mol%) was kept the same in all formulations, which are abbreviated as DOPC, DPPC, and DSPC. Liposome size, dispersity, IJA/ICG, absorption spectra and ICG EE of the prepared liposomes are summarized in **Figure 2**. One-way ANOVA was conducted to compare the effect of liposome membrane rigidity on each of the parameter of the formulations. The difference between the size (F_2,10_ = 2.113, p > 0.05) and dispersity (F_2,10_ = 0.470, p > 0.05) of liposomes were statistically insignificant, with size between 150 - 170 nm with low dispersity (< 0.1) (Figure 2A). However, the effect of bilayer fluidity (DOPC > DPPC > DSPC) was statistically significant on ICG EE (F_2,10_ = 128.8, p < 0.001) (Figure 2B). DOPC-ICG-HBS which exists in the liquid-crystalline phase at room temperature, had an average ICG EE of above 90%, while DPPC-IJA-HBS and DSPC-IJA-HBS, which exist at the liquid-ordered phase at room temperature had an ICG EE of around 25%. Intriguingly, the effect of bilayer fluidity was also statistically significant on IJA/ICG (F_2,10_ = 10.59, p = 0.003). DPPC-IJA-HBS and DSPC-IJA-HBS had IJA/ICG of 2.054 (t_10_ = 2.752, p = 0.020) and 3.292 (t_10_ = 4.602, p < 0.001) compared to the complete absence of IJA in DOPC-HBS formulation (Figure 2C,D). These results indicated that membrane rigidity played an important role in IJA formation regardless of the ICG EE. Based on the highest IJA/ICG value, DSPC formulation was selected for further studies.

### Stable liposomal IJA formulations are successfully prepared in different hydration media

**Figure 3:**
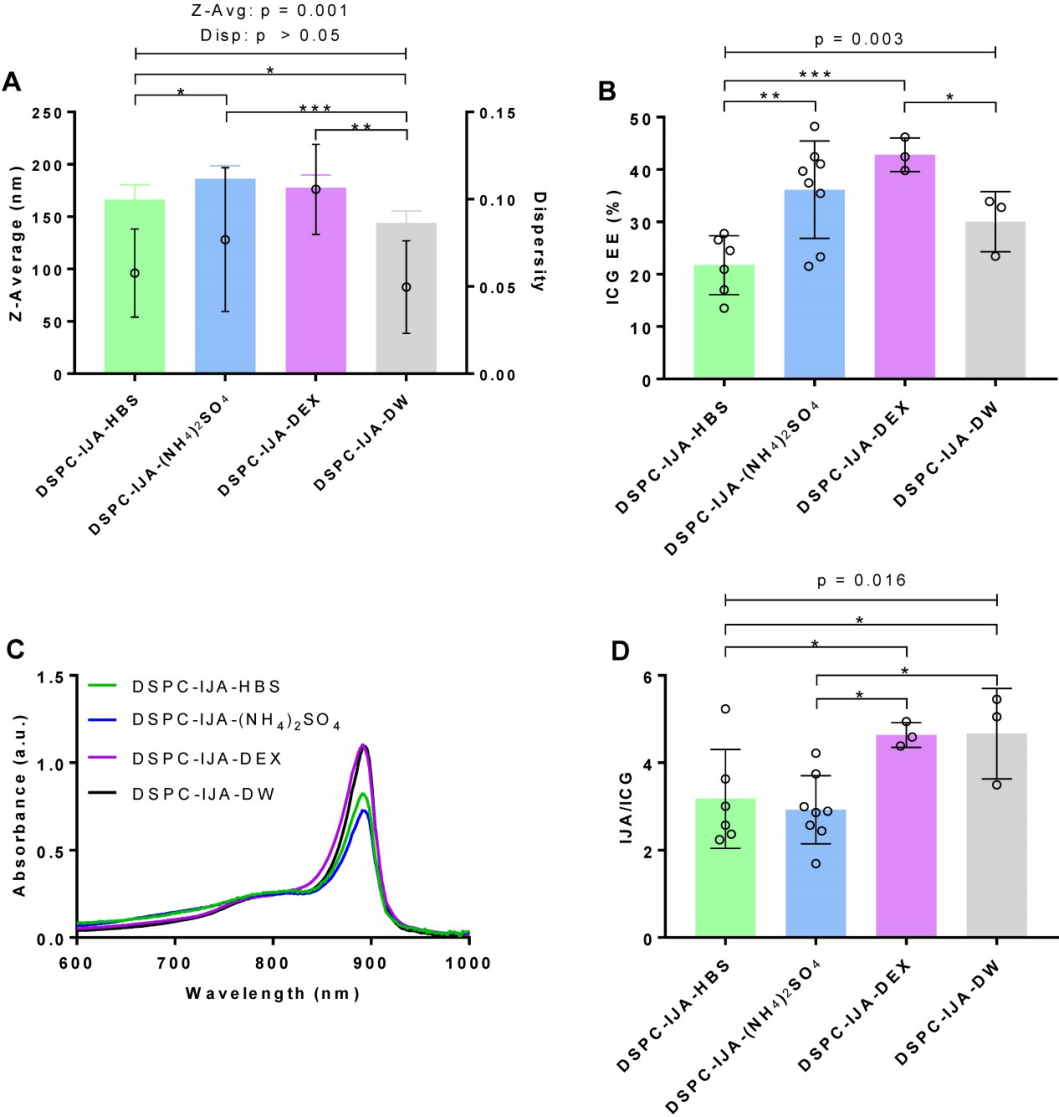
**Characterization of liposomal IJA prepared in different aqueous media.** (A) Z-average diameter and dispersity; (B) ICG EE; (C) absorption spectra; and (D) IJA-to-ICG absorbance ratio of DSPC-IJA-HBS (green), DSPC-IJA-(NH_4_)_2_SO_4_ liposomes (blue), DSPC-IJA-DEX (violet) and DSPC-IJA-DW (gray) liposomes loaded with 180 µM of ICG. Absorption spectra are normalized equivalent to 5 µM of ICG. Data represent mean ± *SD* of at least three independent experiments. *, p < 0.05; **, p < 0.01; ***, p < 0.001; Fisher's LSD test.

Our results confirmed that the solvent environment strongly affects the spectroscopic properties and aggregate states of monomeric ICG, but with minimum effects on IJA (Figure 1). In order to investigate the effect of the hydration medium on our liposomal IJA formation, DSPC-IJA liposomes were prepared in a range of media; namely, HBS, (NH_4_)_2_SO_4_, DEX, and DW. Liposome size, dispersity, IJA/ICG, absorption spectra and ICG EE are presented in **Figure 3** and Table 1. One-way ANOVA was conducted to compare the effect of a hydration medium on each of the parameter of the formulations.

DSPC-IJA-DW formulation was statistically smaller in size (144.2 nm) compared to the liposomal IJA formulations prepared in HBS, (NH_4_)_2_SO_4_ and DEX with 170 - 180 nm (Figure 3A). Nevertheless, dispersity of all prepared liposomes was below 0.1, demonstrating high uniformity regardless of hydration media. The hydration medium had a statistically significant effect on ICG EE (F_3,16_ = 7.125, p = 0.003); with the lowest (21.71%) and highest (42.80%) EE in HBS and DEX media, respectively (Figure 3B). Similarly, IJA/ICG was also significantly affected by the hydration medium (F_3,16_ = 4.634, p = 0.016). DSPC-IJA-DW exhibited the highest IJA/ICG of 4.665, followed by DSPC-IJA-DEX with 4.636, which were significantly different from DSPC-IJA-HBS and DSPC-IJA-(NH_4_)_2_SO_4_ with the ratios of 3.171 and 2.925, respectively (Figure 3C,D). Despite the statistical significances in IJA formation in different media, these results highlight the versatility of our approach to match the hydration medium with the liposomal IJA application, particularly where drug remote loading is required, as will be presented in the next section.

### IJA formation neither affects liposomes morphology/stability nor bilayer integrity

**Figure 4:**
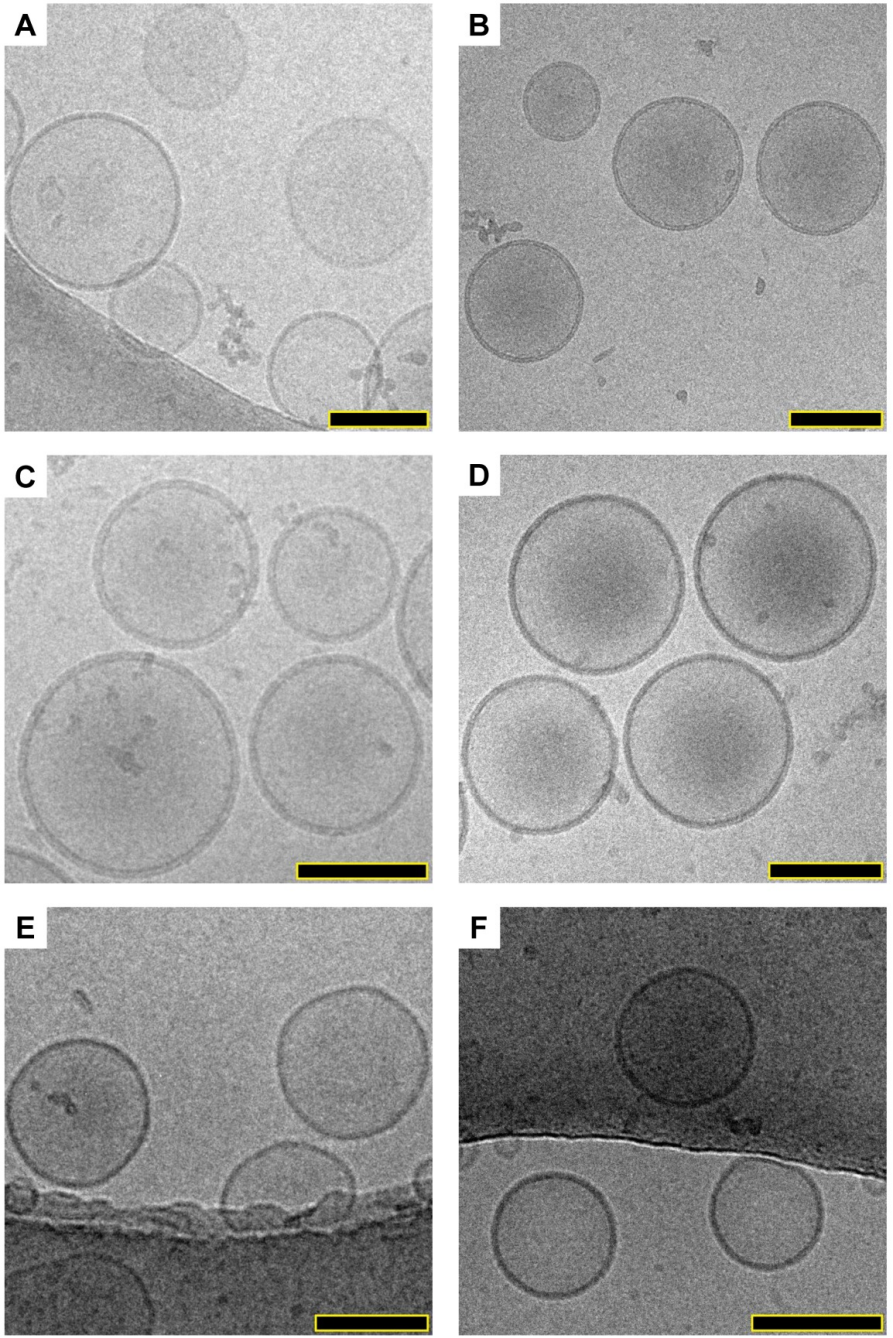
** Structural elucidation of liposomal IJA.** Cryo-TEM micrographs of prepared empty liposomes, liposomal ICG and IJA: (A) DSPC-HBS; (B) DSPC-IJA-HBS; (C) DSPC-IJA-(NH_4_)_2_SO_4_; (D) DSPC-IJA-DW; (E) DOPC-HBS; and (F) DOPC-ICG-HBS. Scale bar: 100 nm.

The morphology of empty liposomes (DOPC- HBS and DSPC-HBS), liposomal ICG (DOPC-IJA- HBS) and IJA (DSPC-IJA-HBS, DSPC-IJA-(NH_4_)_2_SO_4_ and DSPC-IJA-DW) were characterized by cryogenic transmission electron microscopy (cryo-TEM). Interestingly, examining the micrographs (**Figure 4**), all liposomal ICG or IJA retained similar morphology of the empty DOPC or DSPC liposomes. No evident structures could be identified as ICG or IJA, suggesting that IJA formed with the liposomes was very small in size to be imaged by cryo-TEM, compared to free IJA, which could reach hundreds of nanometers in size 21,32. Overall, all IJA liposomes were spherical, and the sizes were in agreement with the DLS data, where DSPC-IJA-(NH_4_)_2_SO_4_ were the largest. Further studies are required to elucidate the shape and the size of liposomes-templated IJA.

The integrity of DSPC-IJA liposome was also assessed by remote loading of doxorubicin using the ammonium sulfate pH-gradient method. The method requires an intact liposome membrane to withhold a pH-gradient, which enables remote loading of doxorubicin (DOX) into the liposome core with high encapsulation efficiency. In DSPC-(NH_4_)_2_SO_4_ co- loaded with both IJA and DOX (DSPC-IJA-(NH_4_)_2_SO_4_- DOX), encapsulation efficiencies for ICG and DOX were 40.37% (*SD* = 6.88%) and 56.63% (*SD* = 9.24%) respectively, demonstrating the integrity of the membrane. Absorption spectra showing the characteristic absorption peaks of IJA (892 nm) and DOX (480 nm) are shown in Figure S4 (Supporting Information).

Finally, a stability study was carried out to investigate the relative spectroscopic change of ICG and IJA (10 µM of ICG equivalent, dispersed in HBS), liposomal ICG (DOPC-ICG-HBS) and liposomal IJA (DPPC-IJA-HBS and DSPC-IJA-HBS). All samples were stored in dark at 4 °C. The stability of the samples was assessed by measuring the absorption spectra over up to 35 days. The relative change in peak absorbance is shown in Figure S5 (Supporting Information). Over 21 days, ICG and IJA were degraded by about 50% and 20% respectively, while liposomal ICG and IJA retained above 90% of their initial absorbance across 35 days, indicating the long-term stability of all our liposomal IJA compared to free IJA.

### Molecular dynamics modeling confirms IJA formation using DSPC lipid bilayer models

Molecular modeling was carried out to evaluate ICG aggregation and IJA formation using lipid bilayers of different fluidity. The initial and equilibrated configurations of ICG in the lipid bilayer models are shown in Figure S6 (Supporting Information). Initially, to examine the degree of alignment of ICG molecule within the clusters formed in the lipid bilayer models, we calculated the orientational order parameter, *O*(*r*) defined as


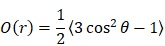


where *r* is the distance between the centers of mass of two ICG molecules and *θ* is the angle between the directions of their longest principal axis. The angle brackets denote ensemble and time average. *O*(*r*) assumes values of 1 for parallel orientation between the examined molecules (θ = 0° or 180°); 0 for random orientation (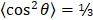); and -0.5 for a perpendicular orientation (θ = 90°). To check for the presence of orientational ordering of ICG molecules within a cluster, only those at short separations, namely up to the distance of the closest neighboring ICG molecules, should be focused on. This distance was determined by the radial distribution function arising from the center of mass of the ICG molecules 33. As shown in Figure S7 (Supporting Information), the distances of ICG molecules from their closest neighbors, which most probably belongs to the same cluster, were approximately up to 10 Å.

**Figure 5:**
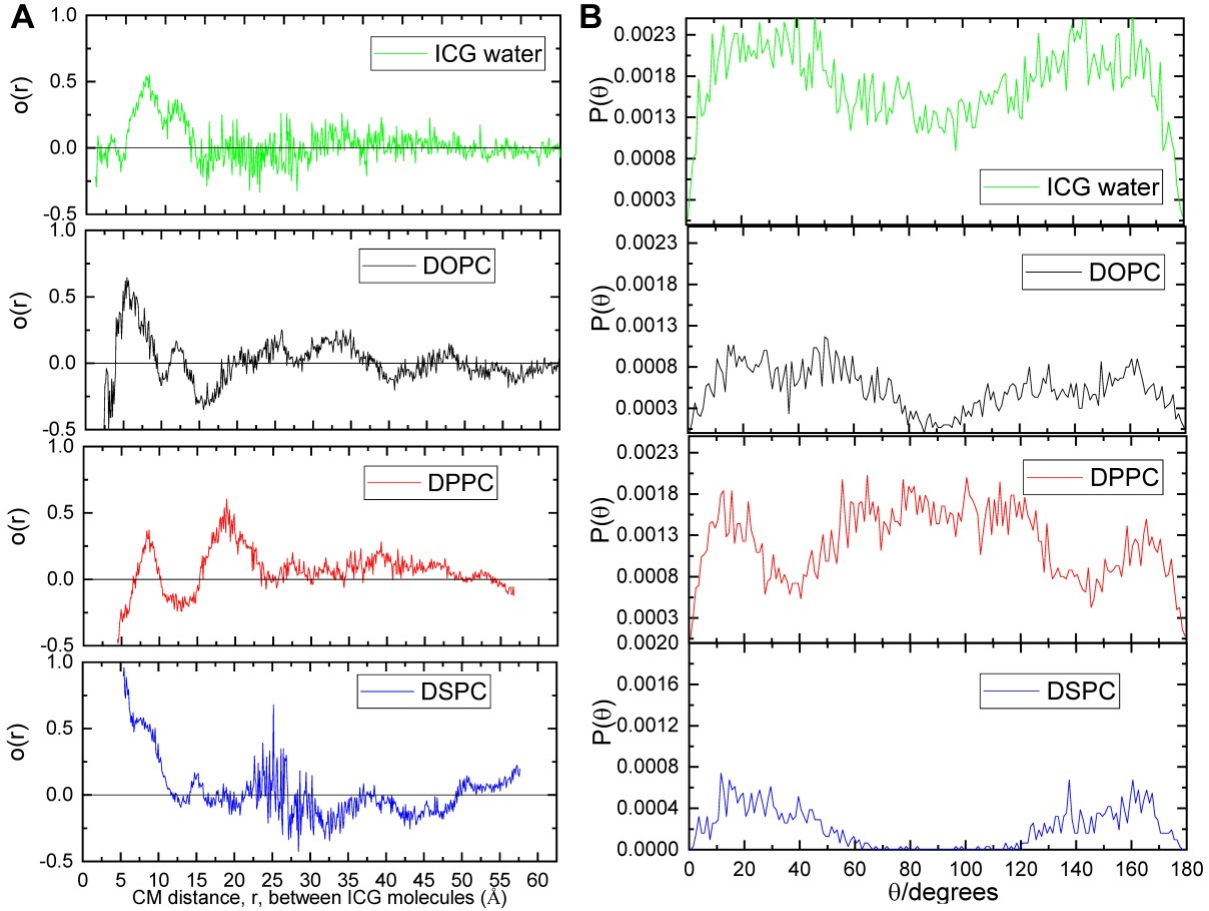
** Orientational order parameter and distribution of angles of the ICG molecules.** (A) Orientational order parameter of the ICG molecules as a function of the distance between their centers of mass. (B) Ensemble and time average of distributions of the angles between the longest axes of ICG molecules in water (green), DOPC (black), DPPC (red) and DSPC (blue) system.

Between the distances of interest (i.e. below 10 Å), the *O*(*r*) values of ICG in water, DOPC, and DPPC systems range between -0.5 to 0.5 with a qualitatively similar manner (**Figure 5**A). In contrast, ICG molecules in the DSPC system always assumed values above 0.5, suggesting a stronger tendency for molecular alignment of ICG for separations below 10 Å. Figure 5B illustrated the distribution of the angles formed by two ICG molecules with separations up to 10 Å. In water, DOPC and DPPC systems, ICG molecules are likely to adopt any orientation between 0° to 180° relative to each other; while in the DSPC system, ICG molecules clearly have greater preference to adopt an almost parallel (θ < 60°) or anti-parallel (θ > 120°) orientation, which agrees with the alignment of J-aggregate, as reported by others 34.

To further elaborate on the characteristics of the formed ICG aggregates, we performed a cluster formation analysis as shown in Figure S8 (Supporting Information) 35. The minimum number of molecules considered to form a cluster was taken to be 2; the maximum separation between two ICG molecules for the detection of the cluster was taken to be 17 Å (in order to include both, first and second neighbors) for all systems; this was inferred from Figure S7 (Supporting Information). The average percentages of ICG molecules participating in clusters were 61%, 47%, 61%, and 61% for water, DOPC, DPPC, and DSPC systems, respectively. In all systems, the majority of the ICG clusters consisted of only 2 molecules (dimer or H-aggregate); only in the DSPC model a five-member cluster has been observed (Figures S8 and S9, Supporting Information), implying a higher tendency for large cluster formation in this system. In conclusion, based on the molecular dynamics modeling results (cluster size, ICG separation distance, and alignment), IJA formation using DSPC lipid bilayer was favored, which agreed with our experimental findings.

### Liposomal IJA but not free IJA restores its NIR fluorescence upon dissociation into monomeric ICG

**Figure 6:**
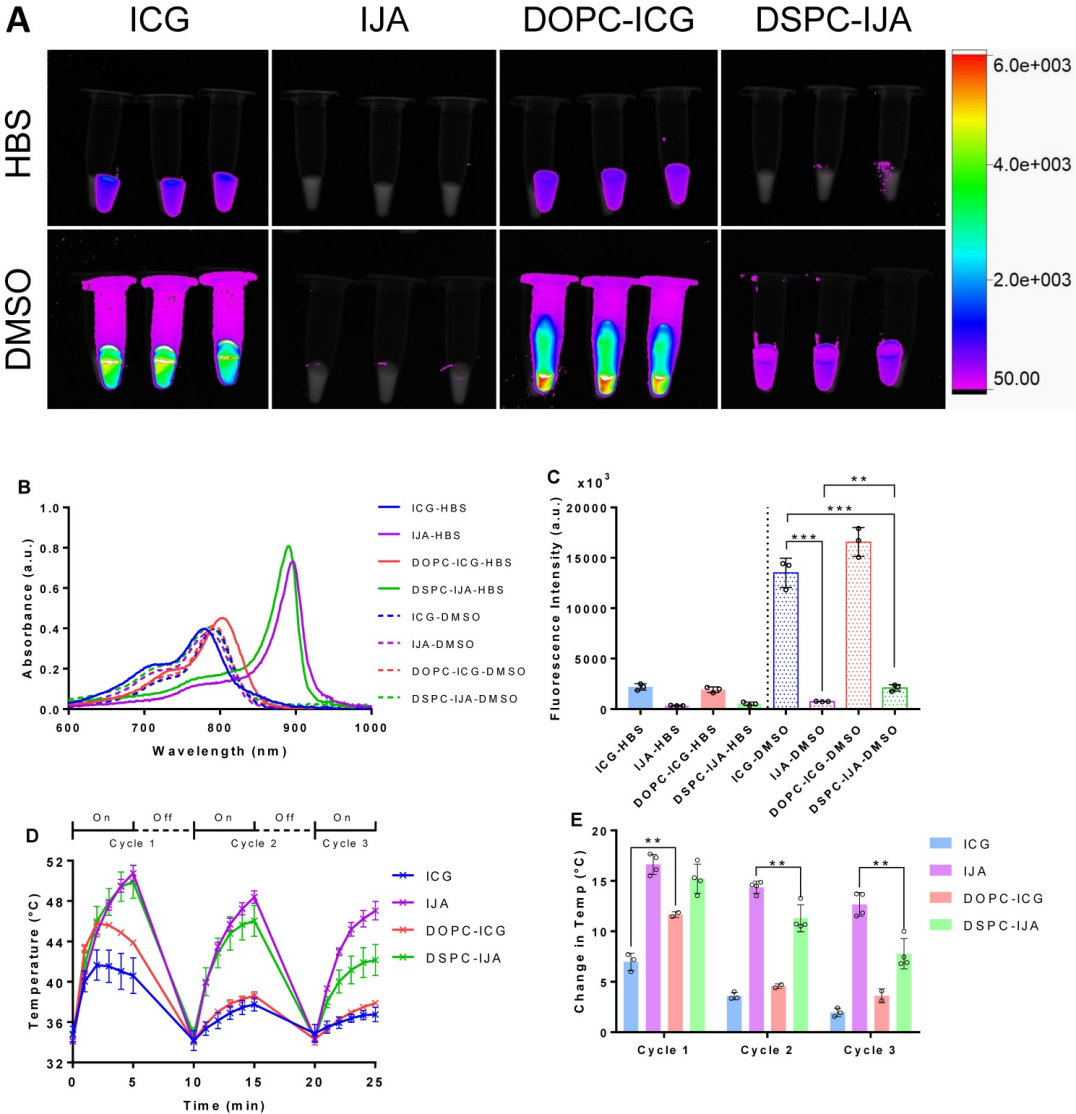
** Fluorescence imaging and photothermal heating of liposomal IJA and liposomal ICG.** (A) Fluorescent images of 2.58 µM of ICG-HBS, IJA-HBS, DOPC-ICG-HBS and DSPC-IJA-HBS dispersed in HBS (top row) and DMSO (bottom row). Samples were imaged with an exposure time of 0.2 s, using excitation and emission wavelengths of 760 nm and 830 nm, respectively. (B) Absorption spectra of ICG-HBS, IJA-HBS, DOPC-ICG-HBS and DSPC-IJA-HBS dispersed in HBS (solid line) and DMSO (dash line). (C) Fluorescence intensity of ICG-HBS, IJA-HBS, DOPC-ICG-HBS and DSPC-IJA-HBS dispersed in HBS (solid bars) and DMSO (dotted bars). (D) Temperature and (E) change in temperature of each heating cycle of ICG-HBS (blue), IJA-HBS (violet), DOPC-ICG-HBS (red) and DSPC-IJA-HBS (green) containing 12.9 µM of ICG. Samples were irradiated with 808 nm laser at 0.5 W cm^-2^. Each sample was irradiated for 5 min and cooled for 5 min; three cycles were performed. Data represent mean ± *SD* of at least three independent experiments. **, p < 0.01; ***, p < 0.001; two-tailed unpaired* t*-tests.

Fluorescence images of free ICG, free IJA, liposomal ICG (DOPC-ICG-HBS formulation) and liposomal IJA (DSPC-IJA-HBS) were taken using excitation and emission wavelengths of 760 nm and 830 nm, respectively. All samples were dispersed in HBS, or in DMSO to dissociate them into monomeric ICG. For samples in HBS, fluorescence signals were detectable only in ICG-containing samples (ICG-HBS and DOPC-ICG-HBS), in contrast to IJA-containing samples (IJA-HBS and DSPC-IJA-HBS) which exhibited minimal fluorescence (**Figure 6**A, top panel). These results were in agreement with their absorption spectra, i.e. IJA absorbs weakly at 760 nm (Figure 6B). The difference between fluorescence intensities of ICG-HBS and DOPC-ICG-HBS was statistically insignificant (t_4_ = 1.135, p > 0.05). For samples dispersed in DMSO, fluorescence intensities were enhanced, especially for ICG-containing samples, due to the increased quantum yield of ICG in DMSO 36. Surprisingly for the IJA-containing samples, despite the increased absorbance at 760 nm with the dissociation of IJA into monomeric ICG (Figure 6B), fluorescence was not restored entirely and remained significantly different (free IJA: t_4_ = 15.13, p < 0.001; DSPC-IJA-HBS: t_4_ = 13.23, p < 0.001) from free ICG which has not undergone J-aggregation **(**Figure 6C); this warrants further investigations.

### Liposomal IJA as a photothermal heating agent

To assess IJA superior photothermal properties, ICG and IJA photothermal stability was examined by three cycles of 808 nm NIR laser irradiation and cooling (Figure 6D,E). During the first cycle, liposomal ICG (DOPC-ICG-HBS) showed a temperature rise of 11.66 °C ± 0.29 °C, which was significantly higher than free ICG with a maximum increase of 6.97 °C ± 0.88 °C (t_3_ = 6.994, p = 0.006). Interestingly, both free ICG and liposomal ICG reached a peak temperature at 2 minutes of the first irradiation cycle, followed by a gradual temperature reduction, which could be attributed to ICG photo-degradation. Subsequent heating cycles were progressively weaker, raising the temperature to around 38 °C, similar to the background heating from HBS (data not shown), suggesting that liposome encapsulation provided ICG with minimum protection against photo-degradation upon irradiation.

In contrast, the peak temperature of free and liposomal IJA (DSPC-IJA-HBS) increased sustainably during the first cycle reaching a maximum of 50.75 °C (Δ*T* = 16.64 °C ± 1.01 °C) and 49.89 °C (Δ*T* = 15.22 °C ± 1.45 °C), respectively*.* Similar increase was observed after the second cycle of heating, however, a slight drop in temperature elevation was observed during the third cycle of laser irradiation (Δ*T*_IJA-HBS_ = 12.66 °C ± 1.18 °C; Δ*T*_DSPC-IJA-HBS_ = 7.79 ± 1.50 °C), which was still greater than the maximum photothermal heating provided by free ICG (at t = 2 min). This confirmed the enhanced photothermal conversion efficiency and thermal stability of IJA, both as a free or liposomal formulation. While the difference between free and liposomal IJA during the first cycle was insignificant (t_4_ = 1.61, p > 0.05), the differences during the second (t_4_ = 4.219, p = 0.006) and the third cycle (t_4_ = 5.123, p = 0.002) was significant. This suggested that while the photothermal heating of free and liposomal IJA was comparable in the first irradiation, liposomal IJA seemed to have a different stability (in solution) against photo-degradation, probably due to the size and structure of the IJA formed in the liposomes, compared to larger and heterogeneous IJA formed in solution, which requires further investigations.

### NIR fluorescent liposomal IJA accumulates in solid tumors following intravenous administration

**Figure 7:**
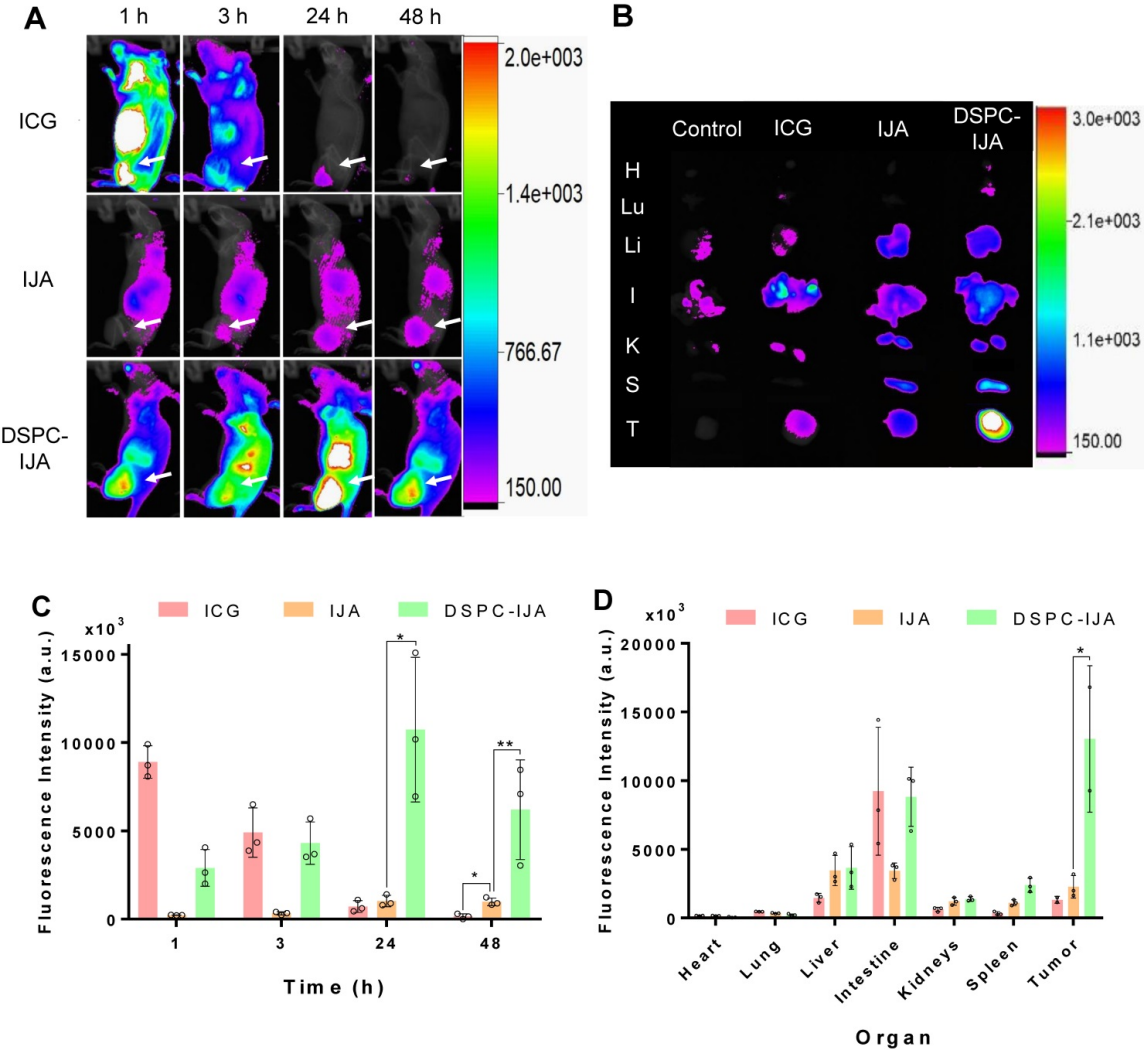
***In vivo* fluorescence imaging and tumor accumulation of liposomal IJA.** (A) In vivo fluorescence images of CT26 tumor-bearing BALB/c mice injected with ICG-HBS (top row), IJA-HBS (middle row) and DSPC-IJA-HBS (bottom row) acquired at 1, 3, 24 and 48 h post injection (left to right column). (B) Ex vivo fluorescence images of major organs and the tumor excised at 48 h post-injection. Abbreviations of organs: H, heart; Lu, lung; Li: liver; I, intestine; K, kidney; S, spleen; and T, tumor. (C) Sum of fluorescent intensity of tumor region of the fluorescence images from (a); n = 3. (D) Sum of fluorescence intensity of major organs and the tumor of the fluorescence images from (B); n = 3. All samples are dispersed in HBS. Data represent mean ± SD of independent experiments. *, p < 0.05; **, p < 0.01; ***, p < 0.001; two-tailed unpaired t-tests, comparing to IJA-HBS of the same time point.

To investigate the *in vivo* biodistribution of DSPC-IJA-HBS, CT26 tumor-bearing BALB/c mice were intravenously injected with ICG, IJA or DSPC-IJA in HBS (0.3 mg kg^-1^) and fluorescence imaging was acquired 1, 3, 24 and 48 h post-injection. It is worth mentioning that same fluorescence scale was used to compare the biodistribution of the three groups over time. Due to the low fluorescence of IJA, the scale threshold was decreased to visualize the IJA signals over time, resulting in extremely high signals in the ICG group. Figure 7A provided qualitative analysis, while Figure 7C & D represented reliable quantitative measurements.

Free ICG exhibited high fluorescence across the whole body 1 h post-injection, followed by quick liver metabolism and biliary excretion (**Figure 7**A,B) 37. A similar trend was observed in CT26 tumors, where strong fluorescence was observed initially (1 h post-injection) followed by a dramatic reduction over time, with complete elimination at 48 h post-injection. This could be explained by ICG presence in tumor vasculature with minimum tumor extravasation and retention 21. On the other hand, free IJA exhibited relatively low but steady fluorescence signals up to 48 h post-injection. Significant accumulation was observed in the liver, intestine and tumor tissues. Although the fluorescence in the tumors was not significantly different from that of free ICG at 24 h post-injection (t_4_ = 1.156, p > 0.05), in agreement with the report by Liu et al. 21, IJA fluorescence was maintained up till 48 h post-injection, which was significantly higher than free ICG (t_4_ = 5.447, p = 0.006) (Figure 7C). In contrast, DSPC-IJA-HBS exhibited an intermediate fluorescence level at 1 h post-injection, with a steady increase in the abdominal region (liver and intestine) and the tumor tissue up to 24 h post-injection. At 48 h time-point, the fluorescence intensity was reduced, with the highest intensity at the tumor site, indicating high tumor retention of the extravasated DSPC-IJA-HBS. Overall, liposomal IJA exhibited greater fluorescence compared to free IJA at all times (Figure 7A), and promisingly, exhibited significantly higher fluorescence intensity at the tumor up to 48 h post-injection (t_4_ = 3.191, p = 0.033) (Figure 7C). Upon organs imaging, IJA and DSPC-IJA-HBS consistently exhibited higher fluorescence intensity in all organs, except the intestine, compared to free ICG, which agrees with its fast metabolism and body elimination. ICG signals in the intestine were highly variable in the ICG group, probably due to the different gastrointestinal transient time between mice. The higher spleen and intestinal accumulation of DSPC-IJA-HBS require further investigations but could be attributed to its higher stability and slower metabolism and elimination from the body, compared to free ICG and IJA (Figure 7D). Promisingly, DSPC-IJA-HBS tumor targeting and imaging were reproduced in 4T1 tumor-bearing BALB/c and C4-2B tumor-bearing NSG mice, following intravenous injection (Figure S10, Supporting Information).

### Liposomal IJA exhibits excellent photothermal stability *in vivo* following multiple laser irradiation

**Figure 8:**
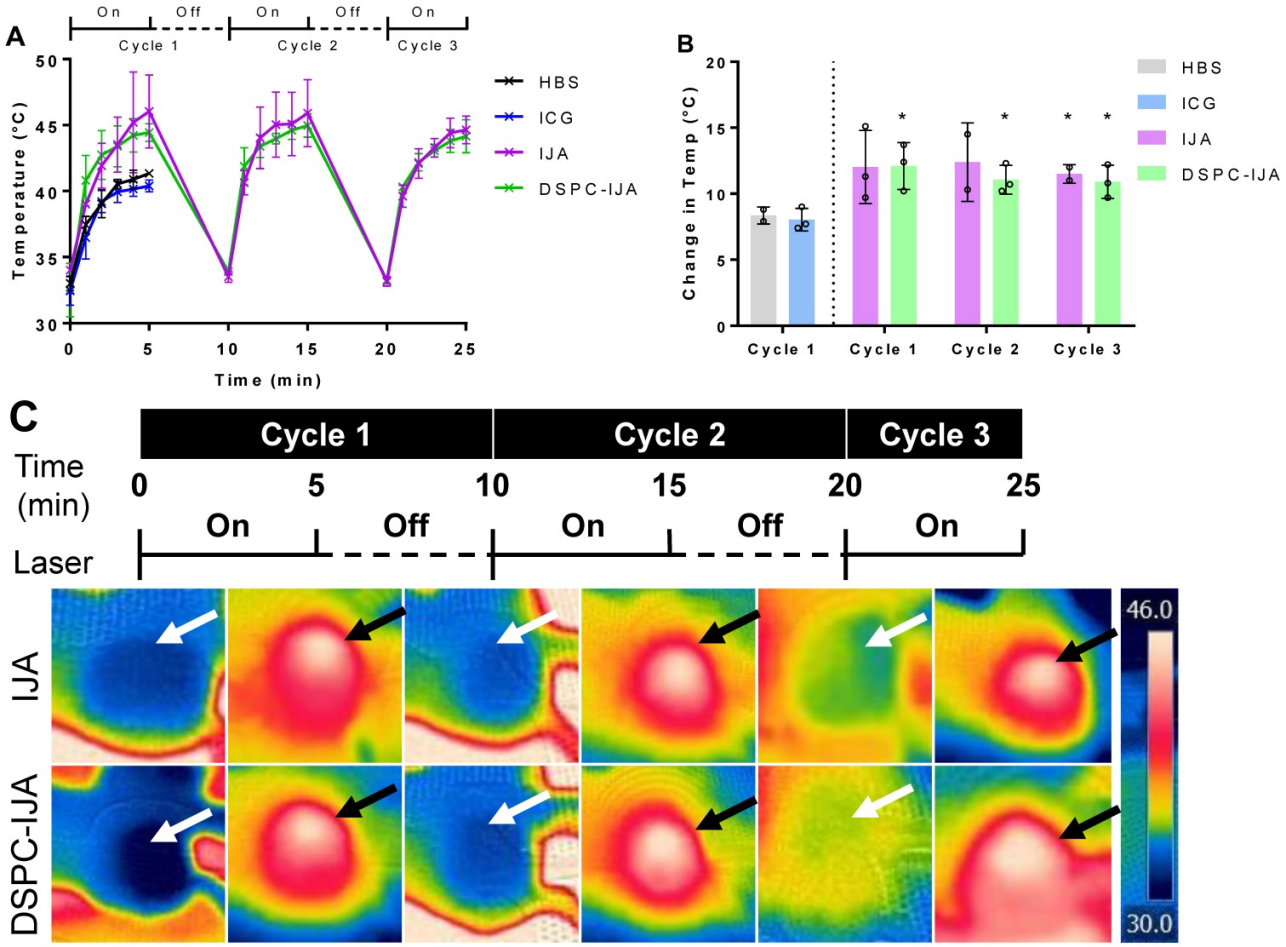
***In vivo* photothermal heating of liposomal IJA.** (A) Temperature and (B) change in temperature of each cycle of mice injected with HBS (black), ICG-HBS (blue), IJA-HBS (violet) and DSPC-IJA-HBS (green). (C) CT26 tumors were irradiated 48 h post- injection with 808 nm laser at 0.5 W cm^-2^. Each tumor was irradiated for 5 min and cooled for 5 min. This cycle was repeated three time for each tumor. Heated and cooled tumors are indicated by white and black arrows, respectively. Data represent mean ± *SD* of independent experiments. *, p < 0.05; two tailed unpaired *t*-tests, comparing to ICG-HBS of cycle 1.

After confirming the efficient liposomal IJA tumor accumulation, 808 nm laser was used for *in vivo* photothermal studies. The laser-induced temperature increase in tumors is shown in **Figure 8**. Photothermal heating in mice injected with HBS, and free ICG similarly reached a maximum temperature of 41 °C. This correlated nicely with the low ICG tumor accumulation 48 h post-injection (Figure 7C); thus, the second and third cycles of laser irradiation were not performed. On the other hand, both free IJA and DSPC-IJA-HBS provided superior photothermal heating, with the maximum temperature increase of 12 °C, reaching up to an average of 46 °C in all three cycles (Figure 8B). In contrast to the *in vitro* photothermal heating results (Figure 6D), where DSPC-IJA-HBS showed decreased photothermal/ heating ability during the second and third cycle, *in vivo* results showed that DSPC-IJA-HBS maintained similar heating stability to free IJA (Figure 8A). As anticipated, photothermal heating of both free IJA (cycle 1: t_4_ = 2.388, p > 0.05; cycle 2: t_3_ = 2.586, p > 0.05; cycle 3: t_3_ = 4.714, p = 0.018) and DSPC-IJA-HBS (cycle 1: t_4_ = 3.588, p = 0.023; cycle 2: t_4_ = 3.785, p = 0.019; cycle 3: t_4_ = 3.279, p = 0.031) were significantly greater than that provided by free ICG (Figure 8B). These promising *in vivo* results highlight the high potential of our liposomal IJA in cancer photothermal therapy using multiple laser irradiation treatment sessions.

## Discussion

J-aggregate high structural stability, in combination with other unique photophysical and spectroscopic properties, led to their application in optoelectronics, chemical, and biological sensing and medical imaging 18. Thus far, indocyanine J-aggregate (IJA) has been prepared using high ICG concentrations (herein 645 µM and reportedly 1.3 - 1.6 mM), in combination with long duration and high temperature (32 h at 65 °C to 1 week at room temperature) 20,23. In the present work, a range of stable, nano-sized liposomal IJA formulations was prepared in different aqueous media, using significantly low initial concentrations of ICG (30 - 180 µM), and within very short period of time (1 - 2 h), which offers a versatile approach for IJA preparation for biomedical applications. Furthermore, liposomal IJA could offer a promising approach for NIR fluorescence-guided drug delivery, optoacoustic imaging, photothermal therapy, and combinatory cancer therapy (photothermal and chemotherapy).

Our unprecedented findings provided a systematic approach to evaluate the parameters (lipid bilayer rigidity, temperature and time) needed for successful nano-liposomal IJA formation. Results showed that IJA formation was significantly affected by the rigidity (or phase behavior) of the lipid bilayer, where the highest IJA level was observed in the rigid DSPC formulation with complete absence in fluidic DOPC liposomes. Similar effects were observed for J-aggregates of pseudoisocyanine (PIC; cyanine dye) 38, and bacteriopheophorbide-lipid (Bchl-lipid; porphyrin dye) 39, where little or no J-aggregates were formed in nanoparticles consisting of unsaturated phospholipids. Mo et al. explained this effect as a surface-mediated templating of monomeric dyes, which could facilitate their intermolecular interaction 38. Thus, only rigid lipid bilayers (low lateral mobility) can enable the formation of the most stable form as J-aggregates. The “template effect” of the ordered bilayer could explain IJA formation exclusively in rigid liposomes even at low ICG concentration. This further agrees with the lower aggregation number of J-aggregates required (of some cyanine dyes) in the presence of lipid bilayers 40. Interestingly, liposomes-templated IJA formation was confirmed, for the first time, using atomistic molecular dynamics. Molecular dynamics simulations suggested that only DSPC lipid bilayer system favors the formation of ICG clusters with almost parallel alignment (Figure 5B); this is essential for formation of J-aggregate, where the stacking angle θ should be less than 54.7° (or otherwise, H-aggregate for 54.7° < θ < 90°) 34.

Up-to-date, several publications have reported ICG loading into non-cholesterol containing, DPPC- or DSPC-based liposomes, without any pieces of evidence of IJA formation 3,11,41. These results are in agreement with our low temperature-sensitive liposomes (DPPC/MSPC/DSPE-PEG_2000_) where no IJA aggregates were formed using 180 µM ICG and 60°C (data not shown). This could be due to the membrane disruption caused by PEG chain insertion into bilayers lacking cholesterol, in the presence of ICG 41; resulting in drug leakage, and disruption of the ordered phospholipid phase essential for J-aggregation. In contrast, our results showed that cholesterol-containing, rigid liposomes promote IJA formation and efficient doxorubicin loading. Currently, there is only one study by Beziere et al. reporting ICG loading into HSPC/Chol/DSPE- PEG_2000_, but with no IJA formation 2. In Beziere et al. study, 208 µM ICG in DEX was used to hydrate the lipid film, yielding ICG-loaded liposomes with a final concentration of 75 µM. The absence of IJA formation, despite the higher ICG concertation used, could be attributed to the passive loading of ICG in the aqueous core, rather than the lipid bilayer. Furthermore, the study did not report the liposome preparation temperature, that could be lower than 60 - 65 °C, which is required for IJA formation.

There are several reports on J-aggregation of short-chained cyanine dye within lipid bilayers, but these formulations were spectroscopically active only in the visible light regions 40,42. Miranda et al. recently reported the first liposomal formulation of J-aggregate, based on a dicarboxylphenyl modified IR-820 dye (DCP-Cy), intended for NIR spleen imaging 43. J-aggregation of DCP-Cy relied on salt-induced J-aggregation, and a basic environment (pH 10 - 11), resulting in an absorbance peak at 934 nm. These harsh conditions reduce the possibilities of other drug loading into the liposomal formulation. Furthermore, during the formation of the DCP-Cy liposomes (by ethanol injection), unidentified yellowish metallic aggregates were developed, instead of the green DCP-Cy color. Interestingly, despite the large liposomes obtained (~450 nm), subsequent size homogenization of the liposomes was excluded to avoid excessive loss (~70%) of the dye, which indicated potential stability issues with the encapsulated material within liposomes. Unsurprisingly, these large DCP-Cy liposomes showed preferential spleen accumulation, most probably due to their large size, which shortened their blood circulation and reduced any potential targeting to organs outside the reticuloendothelial system (RES). In contrast, our liposomal IJA formulations were successfully prepared in a range of hydration media of different ionic strength and pH (DW; HBS, pH 7.4, (NH_4_)_2_SO_4_, pH 5.4), and more promisingly, withstood the extrusion step, and retained their characteristic deep green color. Liposomal IJA were homogeneous, nano-sized (< 200 nm), and capable of tumor passive targeting through enhanced permeability and retention (EPR) effect 44. Moreover, doxorubicin was efficiently loaded in a liposomal formulation close to the clinically-approved Doxil^®^/Caelyx^®^ formulation, offering a new class of theranostics.

IJA has been extensively studied for its spectroscopic and physicochemical properties 6,23. However, only one study has been published evaluating the *in vivo* behavior of free IJA 21. Liu et al. reported promising IJA tumor accumulation and photothermal heating stability upon intravenous administration of a nano-sized IJA. The IJA administered dose was almost 30-fold higher than the dose used in this study (200 µg versus 6 - 9 µg per mouse), most probably, to ensure sufficient fluorescence signal at the selected wavelengths. The unexpected superior *in vivo* fluorescence of our liposomal IJA, compared to free IJA, enabled its tracking *in vivo* at this relatively low ICG dose. Together with extended blood circulation, which could reduce the IJA dose administered for efficient tumor targeting we believe, our liposomal IJA could advance the IJA applications. Since instrumentation and protocols for ICG imaging are already well-established clinically 1, liposomal IJA could be seamlessly translated into the clinic/bedside. Furthermore, exploring the redshift in IJA absorbance and emission could enhance existing imaging modalities 18. Finally, the high thermal stability of liposomal IJA highlights the high potentials of our liposomal IJA as a promising theranostic, offering multi-modal (fluorescence/optoacoustic) imaging, efficient photothermal therapy, and combinatory treatment (chemo- and photothermal therapy).

## Conclusions

A range of liposomal IJA formulations was successfully prepared using the conventional lipid film hydration and extrusion. The liposomal formulation required significantly shorter preparation time and lower ICG concentrations compared to free IJA formation, which is attributed to the “template effect” of lipid bilayer 38. The effect of membrane rigidity, composition, hydration temperature and media on forming liposomal IJA were investigated and discussed. Highly ordered lipid bilayers (e.g. DPPC and DSPC) with a short period of heat treatment (60 - 65 °C) were necessary for liposomal IJA preparation. The engineered liposomal IJA demonstrated prolonged blood circulation, enhanced tumor fluorescence signals, with superior photothermal heating capability. Future work is warranted to assess their applications as an optoacoustic imaging and chemo-photothermal therapy agents.

## Figures and Tables

**Table 1:** Characterization of liposomal formulations prepared in this study.

Formulation	Aqueous Medium	Initial [ICG] (µM)	Z-Avg. (nm)	Dispersity	IJA/ICG	ICG EE (%)
**DSPC**	**HBS**	**0**	147.1 ± 3.8	0.049 ± 0.032	-	-
**DSPC**	**HBS**	**30**	148.0 ± 1.3	0.042 ± 0.028	1.379 ± 0.101	55.78 ± 7.20
**DSPC**	**HBS**	**60**	148.7 ± 2.3	0.032 ± 0.016	2.268 ± 0.339	56.24 ± 11.07
**DSPC**	**HBS**	**120**	145.4 ± 4.6	0.054 ± 0.009	2.547 ± 1.113	39.35 ± 5.30
**DSPC**	**HBS**	**180**	169.9 ± 12.2	0.060 ± 0.028	3.292 ± 1.220	25.78 ± 11.89
**DOPC**	**HBS**	**180**	150.6 ± 5.4	0.076 ± 0.031	0.016 ± 0.009	91.79 ± 9.71
**DPPC**	**HBS**	**180**	155.4 ± 9.2	0.071 ± 0.034	2.054 ± 1.002	23.83 ± 5.26
**DSPC**	**DW**	**180**	144.2 ± 11.4	0.050 ± 0.027	4.665 ± 1.036	30.04 ± 5.74
**DSPC**	**(NH_4_)_2_SO_4_**	**180**	186.1 ± 12.5	0.077 ± 0.041	2.925 ± 0.778	36.15 ± 9.29
**DSPC**	**DEX**	**180**	177.6 ± 12.2	0.106 ± 0.026	4.636 ± 0.282	42.80 ± 3.21

Z-Average diameter, dispersity, IJA-to-ICG absorbance ratio and ICG encapsulation efficiency (EE) of liposomes. Data represents the mean ± *SD* of at least three independent measurements.
