# Supplementary material for: Liposome-Templated Indocyanine Green J- Aggregates for In Vivo Near-Infrared Imaging and Stable Photothermal Heating
Source: Nanotheranostics. 2020 Feb 28;4(2):91–106. doi: 10.7150/ntno.41737 (PMC7064739; doi:10.7150/ntno.41737)
Supplement: Supplementary file 1 — Supplementary figures and tables. [file ntnov04p0091s1.pdf]

Supporting Information

Liposome-Templated Indocyanine Green J- Aggregates for *In Vivo* Near Infrared Imaging and Stable Photothermal Heating

Calvin C. L. Cheung[1], Guanglong Ma[1], Kostas Karatasos[2], Jani Seitsonen[3], Janne Ruokolainen[3], Cédrik-Roland Koffi[1], Hatem A.F.M Hassan[1], and Wafa' T. Al-Jamal[1]*

[1]School of Pharmacy – Queen's University Belfast, Belfast, BT9 7BL, United Kingdom

[2] Department of Chemical Engineering, University of Thessaloniki, P.O. BOX 420, 54124 Thessaloniki, Greece

[3]Department of Applied Physics, Aalto University School of Science, P.O.Box 15100, FI-00076 Aalto, Finland

* Author for correspondence:

Dr. Wafa' T. Al-Jamal

School of Pharmacy, Queen's University Belfast, Belfast, BT9 7BL, United Kingdom.

E-mail: w.al-jamal@qub.ac.uk

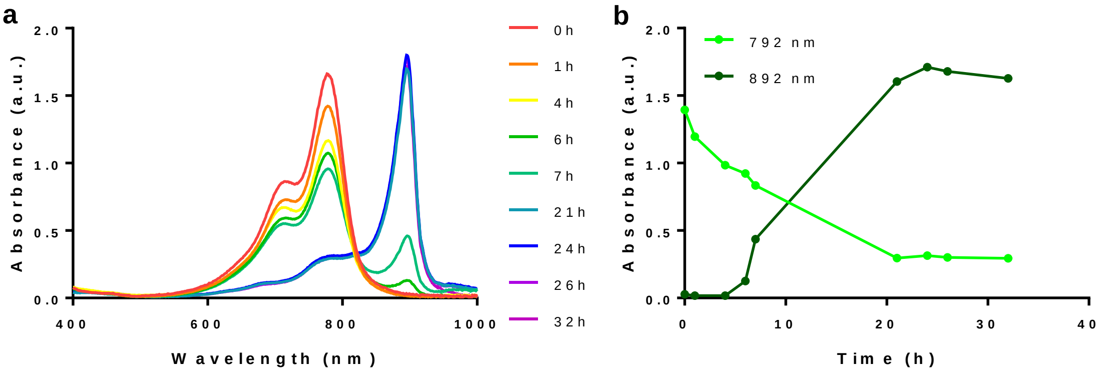

Figure S1. Spectroscopic temporal evolution of IJA formation by heat treatment. (a) Absorption spectra of the temporal evolution of the of ICG into J-aggregates. (b) Temporal evolution of the absorbance at 792 nm (light green) and 892 nm (dark green) of ICG solution. 645 μM of ICG solution in DW was heated at 65 °C for 32 h.

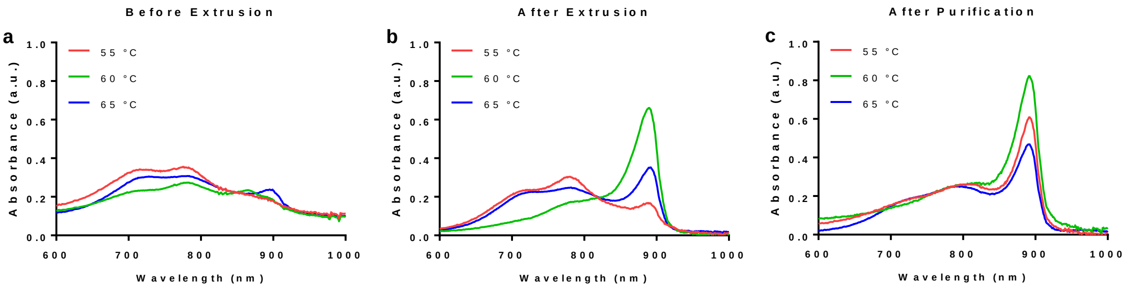

Figure S2. Absorption spectra of DSPC-IJA-HBS liposomes at various stages of preparation. DSPC-IJA-HBS (a) before extrusion, (b) after extrusion and (c) after annealing and purification. The samples were prepared at 55 (red), 60 (green) and 65 °C (blue), loaded with initial ICG concentration of 180 µM. Absorption spectra are normalized equivalent to 5 µM of ICG.

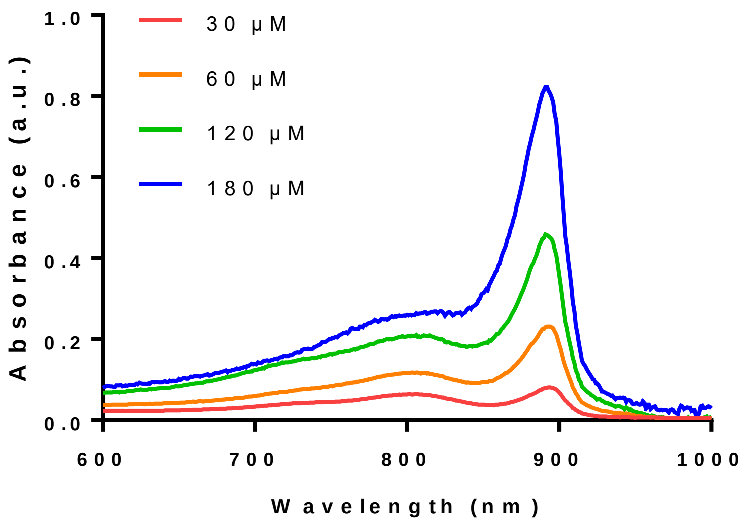

Figure S3. Absorption spectra of liposomal DSPC-IJA-HBS loaded with different ICG concentrations. DSPC-IJA-HBS prepared at 60 °C, loaded with initial ICG concentration of 30 (red), 60 (orange), 120 (green) and 180 μM (blue). Data represents the mean of at least three independent measurements.

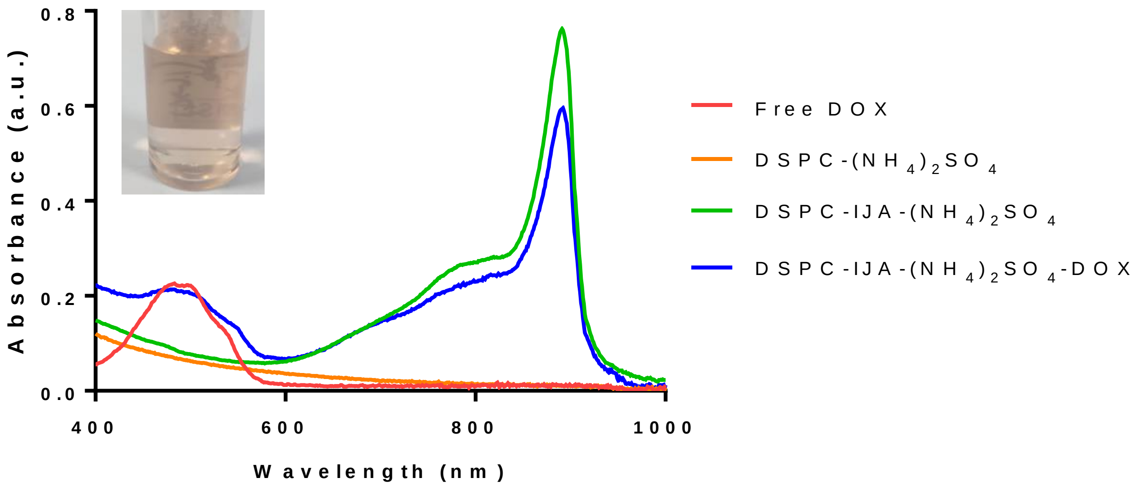

Figure S4. Absorption spectra of DSPC-IJA-$(NH_4)_2SO_4$ co-loaded with DOX. Absorption spectra of free DOX (red), DSPC-$(NH_4)_2SO_4$ (orange), DSPC-IJA-$(NH_4)_2SO_4$ (green) and DSPC-IJA-$(NH_4)_2SO_4$-DOX (blue). The inset represents a photograph of DSPC-IJA-$(NH_4)_2SO_4$-DOX sample. Absorption spectra are normalized equivalent to 5 μM of ICG.

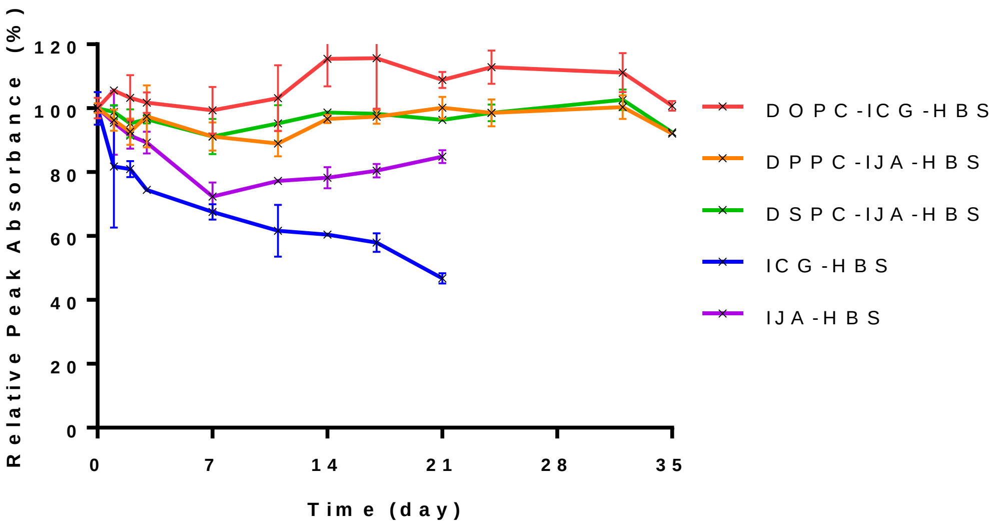

Figure S5. Long-term stability profile of free and liposomal ICG and IJA. Relative peak absorbance of DOPC-ICG (red), DPPC-IJA (orange), DSPC-IJA (green), ICG (blue) and IJA (violet) up to 35 days compared to the initial (t = 0 day) value. Samples were dispersed in HBS, stored in dark at 4 °C during the stability study. ICG-HBS and IJA-HBS contained equivalent to 10 µM of ICG. Data represent the mean ± *SD* of at least three independent measurements.

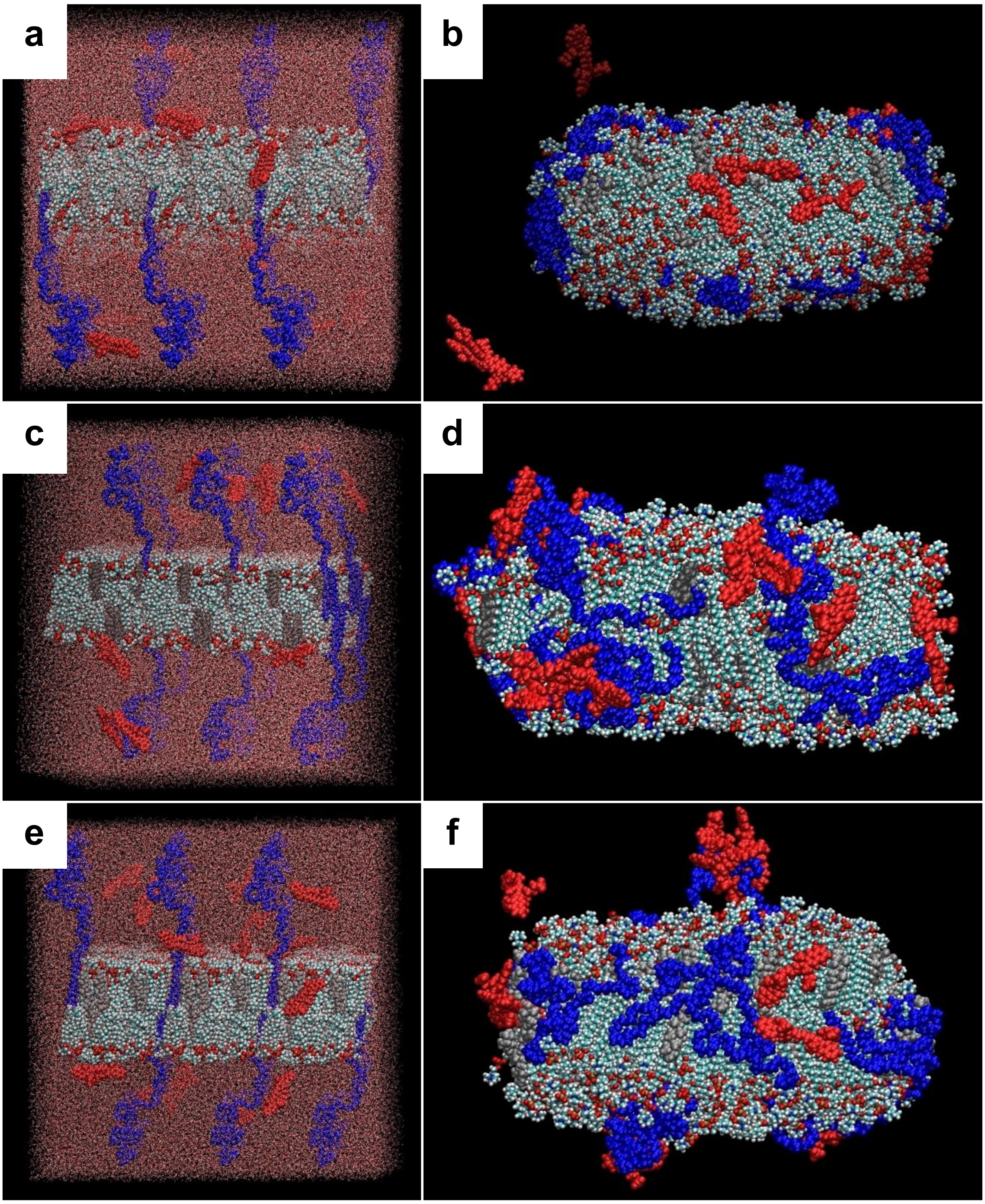

Figure S6. Snapshots of the initial configurations (left) and the equilibrated (right) lipid bilayer models. Space filling molecular models of (a-b) DOPC, (c-d) DPPC and (e-f) DSPC lipid bilayer; ICG in red; DSPE-PEG$_{2000}$ in blue; cholesterol in gray; phospholipid in cyan/red/white; and water molecules in red/white. Water molecules are omitted in the equilibrated configuration (b; d; f) for clarity.

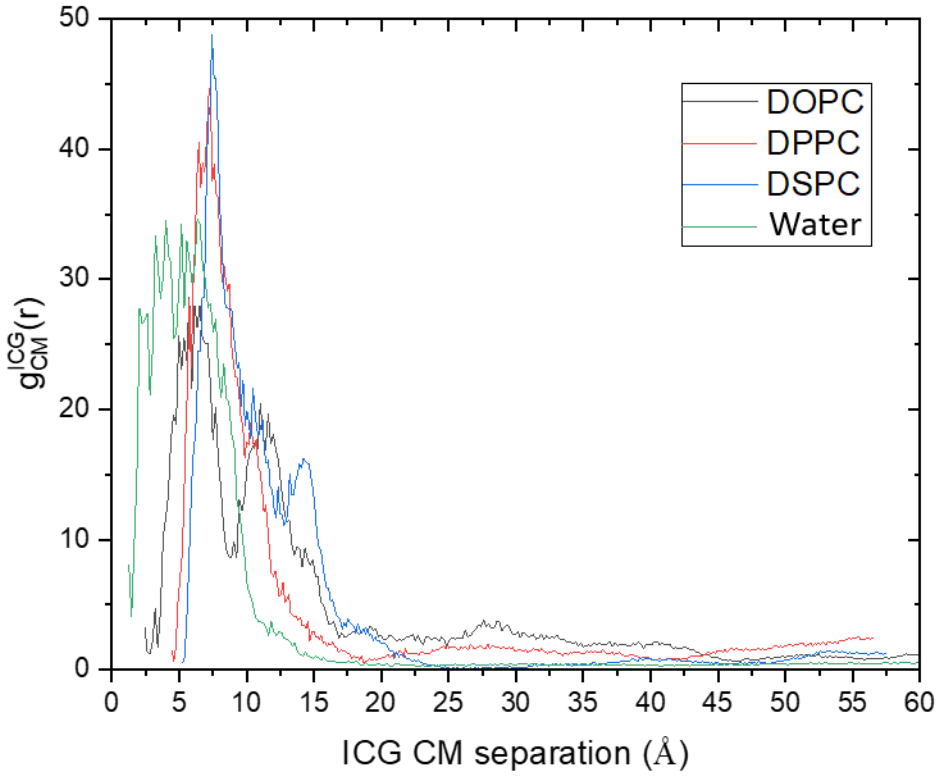

Figure S7. Radial distribution function for the ICG molecules in the examined systems. Radial distribution function of the center of mass of the ICG molecules in water (green), DOPC (black), DPPC (red) and DSPC (blue) systems. Values are normalized to the same number density.

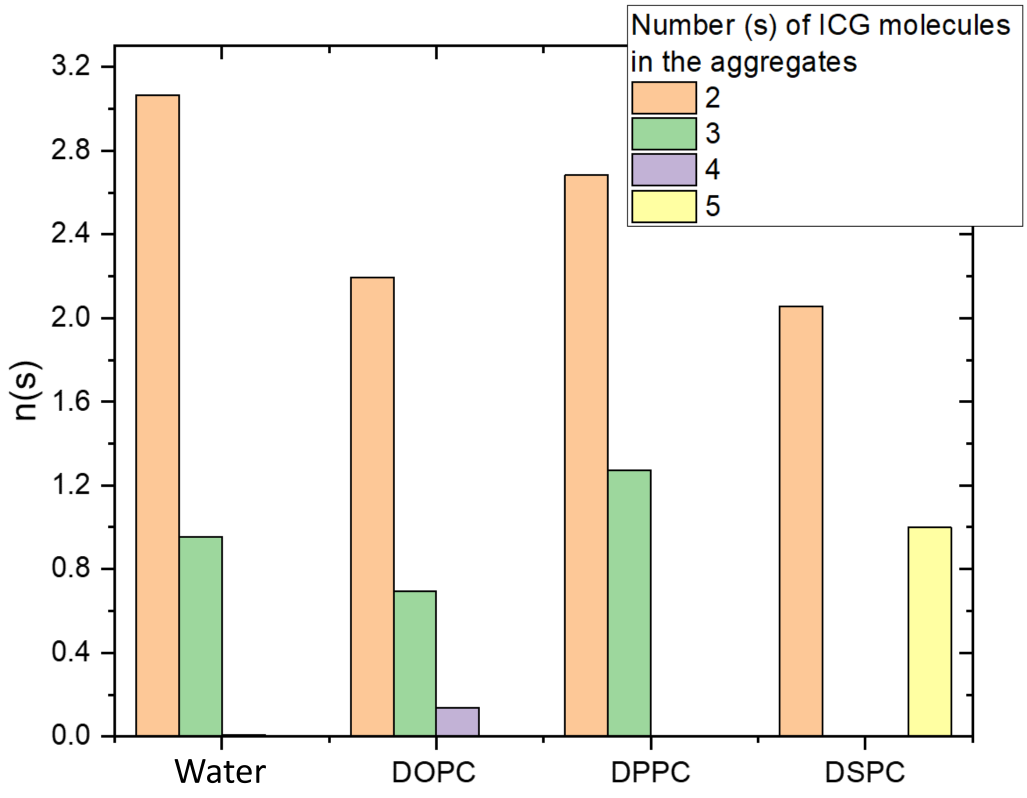

Figure S8. **Cluster formation analysis on ICG in the four examined models**. Frame-averaged number of ICG cluster against the cluster size in water, DOPC, DPPC, and DSPC systems. Cluster consisted of two (orange), three (green), four (violet), five (yellow) ICG molecules. Where bars are missing, the corresponding values lay within the error margin.

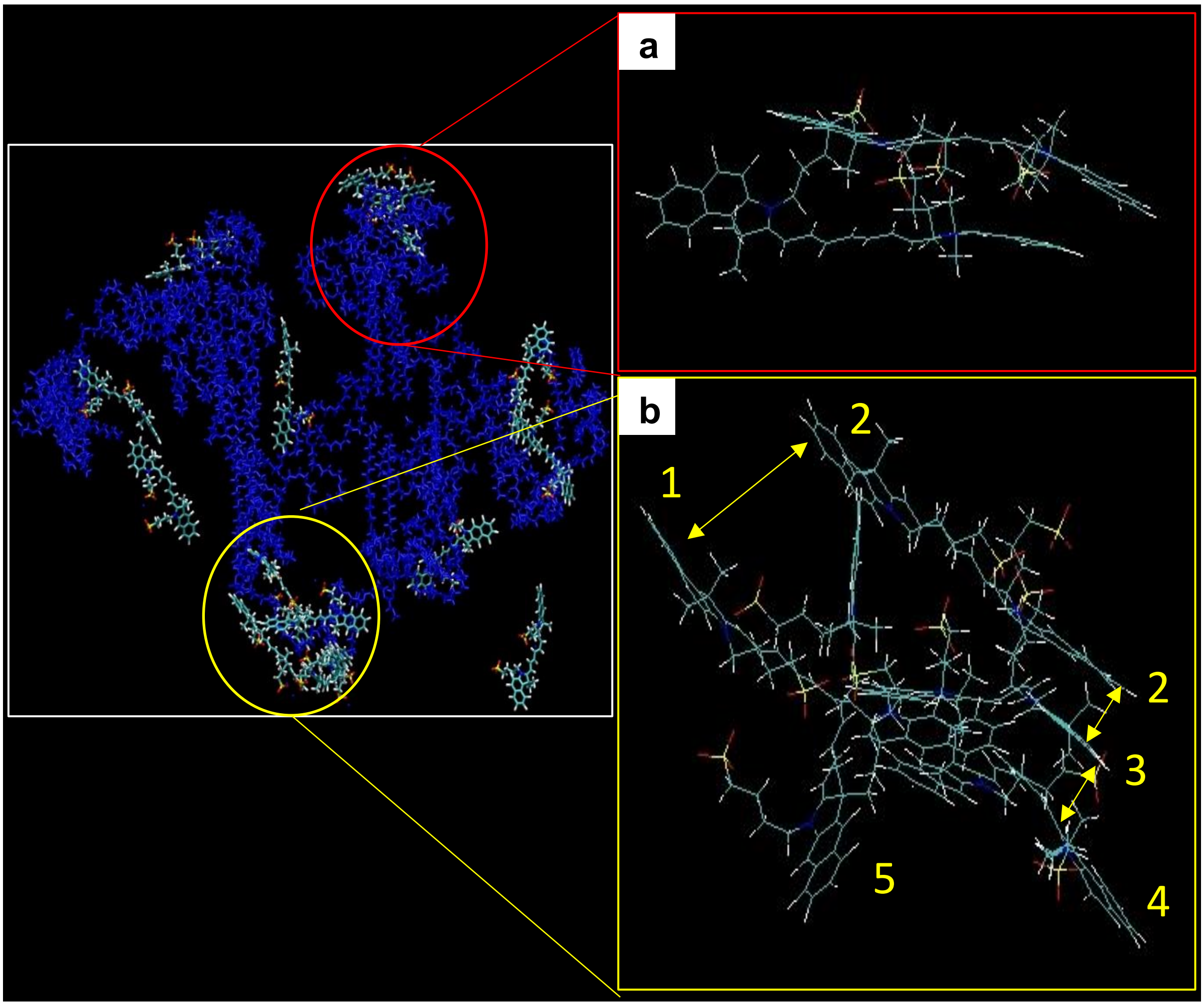

Figure S9. **Depiction of ICG aggregate (IJA) formation in the DSPC bilayer model**. Snapshots of (a) a two-member cluster with parallel/aligned relative orientations; and (b) a five-member cluster, which the pairs (1,2), (2,3) (3,4) and the triplet (2,3,4) assume almost parallel relative orientations. DSPE-PEG$_{2000}$ are shown in blue, while all other molecular components are omitted for clarity.

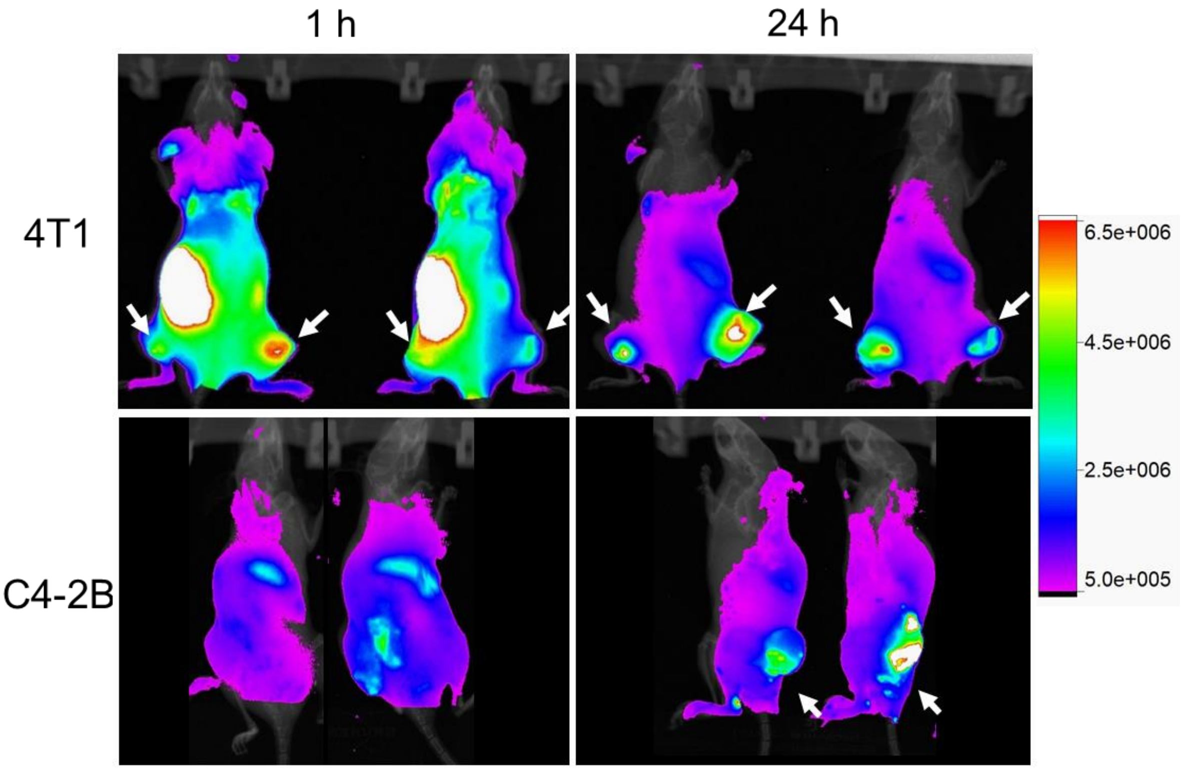

Figure S10. Tumor accumulation of DSPC-IJA-HBS following intravenous administration. *In vivo* fluorescence images of 4T1 tumor-bearing BALB/c mice (top row), and C4-2B tumor-bearing NSG mice (bottom row) injected intravenously with DSPC-IJA-HBS at 0.45 mg kg^{-1} and 0.3 mg kg^{-1}, respectively. The images were taken at 1 h (left) and 24 h (right) post-injection. Tumors are indicated by white arrows.
